# A *Trans*-Ethnic Genome-Wide Association Study of Uterine Fibroids

**DOI:** 10.3389/fgene.2019.00511

**Published:** 2019-06-12

**Authors:** Todd L. Edwards, Ayush Giri, Jacklyn N. Hellwege, Katherine E. Hartmann, Elizabeth A. Stewart, Janina M. Jeff, Michael J. Bray, Sarah A. Pendergrass, Eric S. Torstenson, Jacob M. Keaton, Sarah H. Jones, Radhika P. Gogoi, Helena Kuivaniemi, Kathryn L. Jackson, Abel N. Kho, Iftikhar J. Kullo, Catherine A. McCarty, Hae Kyung Im, Jennifer A. Pacheco, Jyotishman Pathak, Marc S. Williams, Gerard Tromp, Eimear E. Kenny, Peggy L. Peissig, Joshua C. Denny, Dan M. Roden, Digna R. Velez Edwards

**Affiliations:** ^1^Division of Epidemiology, Department of Medicine, Vanderbilt University Medical Center, Nashville, TN, United States; ^2^Vanderbilt Epidemiology Center, Institute for Medicine and Public Health, Vanderbilt University Medical Center, Nashville, TN, United States; ^3^Vanderbilt Genetics Institute, Vanderbilt University Medical Center, Nashville, TN, United States; ^4^Division of Quantitative Sciences, Department of Obstetrics and Gynecology, Vanderbilt University School of Medicine, Nashville, TN, United States; ^5^Division of Reproductive Endocrinology and Infertility, Departments of Obstetrics and Gynecology and Surgery, Mayo Clinic, Rochester, MN, United States; ^6^Charles Bronfman Institute for Personalized Medicine, Icahn School of Medicine at Mount Sinai, New York, NY, United States; ^7^Biomedical and Translational Informatics Institute, Geisinger Health System, Danville, PA, United States; ^8^Sigfried and Janet Weis Center for Research, Geisinger Health System, Danville, PA, United States; ^9^SAMRC-SHIP South African Tuberculosis Bioinformatics Initiative, Division of Molecular Biology and Human Genetics, Department of Biomedical Sciences, Faculty of Medicine and Health Sciences, Stellenbosch University, Stellenbosch, South Africa; ^10^Center for Health Information Partnerships, Department of Medicine, Feinberg School of Medicine, Northwestern University, Chicago, IL, United States; ^11^Department of Medicine, Feinberg School of Medicine, Northwestern University, Chicago, IL, United States; ^12^Department of Cardiovascular Diseases, Mayo Clinic, Rochester, MN, United States; ^13^Department of Family Medicine and Behavioral Health, University of Minnesota Medical School, Duluth, MN, United States; ^14^Department of Medicine, University of Chicago, Chicago, IL, United States; ^15^Center for Genetic Medicine, Feinberg School of Medicine, Northwestern University, Chicago, IL, United States; ^16^Division of Health Informatics, Department of Healthcare Policy and Research, Weill Cornell Medicine, New York, NY, United States; ^17^Genomic Medicine Institute, Geisinger, Danville, PA, United States; ^18^Center for Statistical Genetics, Icahn Institute for Genomics and Multiscale Biology, Icahn School of Medicine at Mount Sinai, New York, NY, United States; ^19^Biomedical Informatics Research Center, Marshfield Clinic Research Institute, Marshfield, WI, United States; ^20^Department of Biomedical Informatics and Department of Medicine, Vanderbilt University Medical Center, Nashville, TN, United States; ^21^Departments of Medicine, Pharmacology, and Biomedical Informatics, Vanderbilt University Medical Center, Nashville, TN, United States

**Keywords:** uterine fibroids, genome-wide association study (GWAS), *trans*-ethnic, meta-analysis electronic health record (EHR), genetically predicted gene expression (GPGE), genetic architecture

## Abstract

Uterine fibroids affect up to 77% of women by menopause and account for up to $34 billion in healthcare costs each year. Although fibroid risk is heritable, genetic risk for fibroids is not well understood. We conducted a two-stage case-control meta-analysis of genetic variants in European and African ancestry women with and without fibroids classified by a previously published algorithm requiring pelvic imaging or confirmed diagnosis. Women from seven electronic Medical Records and Genomics (eMERGE) network sites (3,704 imaging-confirmed cases and 5,591 imaging-confirmed controls) and women of African and European ancestry from UK Biobank (UKB, 5,772 cases and 61,457 controls) were included in the discovery genome-wide association study (GWAS) meta-analysis. Variants showing evidence of association in Stage I GWAS (*P* < 1 × 10^-5^) were targeted in an independent replication sample of African and European ancestry individuals from the UKB (Stage II) (12,358 cases and 138,477 controls). Logistic regression models were fit with genetic markers imputed to a 1000 Genomes reference and adjusted for principal components for each race- and site-specific dataset, followed by fixed-effects meta-analysis. Final analysis with 21,804 cases and 205,525 controls identified 326 genome-wide significant variants in 11 loci, with three novel loci at chromosome 1q24 (sentinel-SNP rs14361789; *P* = 4.7 × 10^-8^), chromosome 16q12.1 (sentinel-SNP rs4785384; *P* = 1.5 × 10^-9^) and chromosome 20q13.1 (sentinel-SNP rs6094982; *P* = 2.6 × 10^-8^). Our statistically significant findings further support previously reported loci including SNPs near *WT1, TNRC6B, SYNE1, BET1L*, and *CDC42*/*WNT4*. We report evidence of ancestry-specific findings for sentinel-SNP rs10917151 in the *CDC42*/*WNT4* locus (*P* = 1.76 × 10^-24^). Ancestry-specific effect-estimates for rs10917151 were in opposite directions (P-Het-between-groups = 0.04) for predominantly African (OR = 0.84) and predominantly European women (OR = 1.16). Genetically-predicted gene expression of several genes including *LUZP1* in vagina (*P* = 4.6 × 10^-8^), *OBFC1* in esophageal mucosa (*P* = 8.7 × 10^-8^), *NUDT13* in multiple tissues including subcutaneous adipose tissue (*P* = 3.3 × 10^-6^), and *HEATR3* in skeletal muscle tissue (*P* = 5.8 × 10^-6^) were associated with fibroids. The finding for *HEATR3* was supported by SNP-based summary Mendelian randomization analysis. Our study suggests that fibroid risk variants act through regulatory mechanisms affecting gene expression and are comprised of alleles that are both ancestry-specific and shared across continental ancestries.

## Introduction

Uterine leiomyomata, also known as fibroids, are the most common female pelvic tumor and are the indication for at least 37% of all hysterectomies, or approximately 200,000 hysterectomies annually in the United States (Cramer and Patel, 1990; Wu et al., 2007). The estimated financial burden in the United States ranges from $9.4 billion per year in direct costs, to $34.4 billion per year considering all costs(Cardozo et al., 2012). Fibroid prevalence estimates range from 20 to 80%, increasing with age up to menopause (Cramer and Patel, 1990; Marshall, 1997; Vollenhoven, 1998).

Known risk factors for fibroids include African ancestry, African American (AA) race (Ojeda, 1979; Cramer and Patel, 1990; Marshall, 1997; Faerstein et al., 2001a,b; Baird et al., 2003), early age-at-menarche (Lumbiganon et al., 1996; Samadi et al., 1996; Marshall et al., 1998; Faerstein et al., 2001a; Wise et al., 2004; Dragomir et al., 2010), high body mass index (BMI) (Moore et al., 2008; Takeda et al., 2008; Stewart et al., 2017), and increasing age up to menopause (Baird et al., 2003). Higher parity is inversely associated with fibroids, likely due to pregnancy-related hormonal and physical changes including postpartum uterine involution (Baird and Dunson, 2003; Laughlin et al., 2010, 2011).

Multiple lines of evidence suggest fibroids are influenced by genetic risk factors. Twin and familial aggregation studies in several European populations estimate heritability of fibroids between 26 and 69% (Kurbanova et al., 1989; Snieder et al., 1998; Luoto et al., 2000). Additionally, the observations that AA women develop fibroids at an earlier age, have more numerous and larger fibroids, and have a higher lifetime incidence of fibroids further suggest a genetic contribution to fibroid risk (Baird et al., 2003). Various growth factors (Sozen and Arici, 2002), reproductive factors (Parazzini et al., 1996), dysregulation of microRNAs (Marsh et al., 2008), shortening of telomeres (Bonatz et al., 1998), excessive production of disorganized extracellular matrix (Sozen and Arici, 2002; Malik et al., 2010), and acquired chromosomal aberrations have also been noted as potential factors contributing to the development of fibroids (El-Gharib and Elsobky, 2010).

Cha et al. (2011a) used a two-stage case-control genome-wide association study (GWAS) to examine risk for fibroids in a population of Japanese women from hospitals affiliated with the BioBank Japan Project. They reported genome-wide significant single nucleotide polymorphisms (SNPs) in three chromosomal regions, 10q24.33, 11p15.5, and 22q13.1, corresponding to nearby genes *OBFC1, BET1L*, and *TNRC6B*, respectively. The associations at *BET1L* and *TNRC6B* were previously replicated in our imaging-confirmed case-control study of white women from the Vanderbilt University biorepository (BioVU) (Edwards et al., 2013b).

Eggert et al. (2012) performed a large-scale genome-wide linkage scan (GWLS) reporting discoveries in chromosomes 10p11 and 3p21. They reported a genome-wide significant result for rs4247357, an intronic SNP in *CCDC57*, from self-reported case-control investigations from the Women’s Genome Health Study and the Australian Cohort Association Study. However, the regions identified in the Japanese GWAS (Cha et al., 2011a), the Australian GWLS and the European ancestry GWAS do not have any notable overlaps.

We have also previously published a GWAS of fibroid risk in recent African ancestry populations, as well as an admixture mapping study of fibroid risk considering interactions with BMI (Giri et al., 2017; Hellwege et al., 2017a). In those studies the *CYTH4* (chromosome 22q13.1) locus was detected in African ancestry populations (Hellwege et al., 2017a), as well as the *ADTRP* (chromosome 6p24) and *TFPI* (chromosome 2q 31-32) loci for ancestry-BMI interactions (Giri et al., 2017).

Two recent GWASs have combined data from white British participants from the United Kingdom Biobank (UKB) with Icelandic (Rafnar et al., 2018) and Finnish (Välimäki et al., 2018) fibroid cases and controls. These studies reported variants in 16 and 22 loci, respectively, to be associated with uterine fibroids (Rafnar et al., 2018; Välimäki et al., 2018). Both studies replicated the previously reported loci from the Japanese study, *OBFC1, BET1L*, and *TNCR6B*, as well as both identifying several novel loci, including *TP53, TERT, ATM, CDC42*/*WNT4*, and *SYNE1*/*ESR1*. These studies also used males as controls, which may bias to the null hypothesis when male controls carry risk-increasing alleles but they do not have a uterus in which to grow fibroids, or may induce spurious associations.

Despite the high prevalence of fibroids and strong racial disparities between European Americans (EA)s and AAs, no trans-ethnic GWAS meta-analysis combining AA and EA women has been conducted. We performed a two-stage trans-ethnic GWAS of uterine fibroid risk in women of predominantly African and European ancestries in the electronic Medical Records and Genomics (eMERGE) network and the UKB (McCarty et al., 2011; Gottesman et al., 2013) to identify both shared and unique genetic variants associated with fibroid risk across ancestry groups.

## Materials and Methods

### Study Population

**FIGURE 1:**
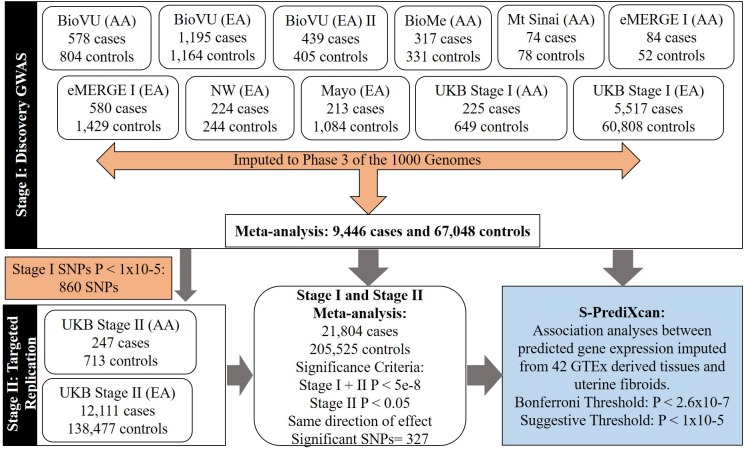
Flow-chart showing study stages and types of analyses.

Stage I GWAS analyses included fibroid cases and female controls from eleven European and African ancestry-specific subsets, nine identified from sites in the eMERGE network and two identified from African and European subsets of the UKB from the 150K release. eMERGE sites included the BioVU Biobank at Vanderbilt University (BioVU), the Mayo Clinic, Mount Sinai, Marshfield Clinic, Northwestern University, and Group Health (Table 1 and Figure 1). A detailed description of the structure and organization of the eMERGE network has been previously published (McCarty et al., 2011). Clinical data from eMERGE cohorts rely on multiple sources including diagnostic and procedure codes, basic demographics, discharge summaries, progress notes, health history, multi-disciplinary assessments, laboratory values, imaging reports, medication orders, and pathology reports. The Institutional Review Boards at all participating institutions in the eMERGE network approved this study. Genotype data utilized in this study from BioVU and the eMERGE network are publically available in NCBI dbGaP, accession numbers phs001409.v1.p1 and phs000360.v2.p1, respectively. Data from UK Biobank may be obtained by qualified investigators upon application with UK Biobank.

**Table 1:** Race- and site-specific characteristics of women across participating eMERGE sites by fibroid case-control status.

Study	Race	Cases	Controls	Age Mean (*SD*) Cases	Age Mean (*SD*) Controls	*P*	BMI Mean (*SD*) Cases	BMI Mean (*SD*) Controls	*P*
BioVU	AA	578	804	40 (11)	41 (15)	0.150	31 (14)	26 (16)	<0.0001
BioVU	EA	1,195	1,164	46 (12)	56 (19)	<0.0001	27 (11)	25 (12)	<0.0001
BioVU-II	EA	439	405	47 (12)	49 (16)	0.04	28 (10)	25 (13)	0.0002
Mount Sinai: Bio*Me*^TM^	AA	317	331	48 (10)	45 (16)	0.004	32 (9)	32 (10)	0.999
Mount Sinai	AA	74	78	59 (10)	63 (12)	0.027	NA	NA	NA
eMERGE I (Northwestern)	AA	84	52	47 (9)	31 (4)	<0.0001	33 (9)	31 (8)	0.178
eMERGE I (Marshfield/NU/Group Health)	EA	580	1,429	53 (11)	68 (12)	<0.0001	29 (6)	28 (6)	0.0007
Northwestern	EA	224	244	50 (10)	37 (8)	<0.0001	26 (7)	26 (6)	0.999
Mayo Clinic	EA	213	1,084	55 (11)	65 (11)	<0.0001	31 (18)	29 (9)	0.115
UKB	African	225	649	50 (6)	54 (7)	0.002	30 (6)	30 (6)	0.229
UKB	European	5,517	60,808	56 (8)	58 (7)	<0.0001	28 (5)	27 (5)	<0.0001
Stage I Total		9,446	67,048						
UKB – Replication	African	247	713	49 (7)	54 (7)	<0.001	31 (6)	31 (6)	0.999
UKB – Replication	European	12,111	137,764	57 (8)	58 (7)	<0.001	28 (5)	27 (5)	<0.0001
Stage II Total		12,358	138,477						
Total		21,804	205,525						


*AA, African American; BioVU, Bio-repository at Vanderbilt University; BMI, body mass index; EA, European American; eMERGE, electronic Medical Records and Genomics; eMERGE I (Marshfield/NU/Group Health), Participants from these sites were genotyped with the same chip, harmonized together and analyzed as one set; SD, standard deviation; NA, Not Available; NU, Northwestern University; UKB, UK Biobank.*

The UKB is a large long-term biobank study in the United Kingdom started in 2006, capable of investigating the role of multiple environmental and genetic factors associated with disease development. The study enrolled 499,589 adults 40–69 years of age in 2006 and the planned follow-up is for 30 years since enrollment.

There were two releases of the UKB genetic dataset; the first release included 150K individuals and the second release included the entire 500K individuals in the study. Stage I of this study utilized European and African-ancestry samples in the first release, and Stage II included European and African-ancestry samples from the second release, excluding individuals from the first release to provide independent replication sets.

### Fibroid Diagnosis for eMERGE Cohorts

A detailed description and validation of our fibroid phenotyping algorithm has been published (Feingold-Link et al., 2014). Briefly, the algorithm uses a combination of demographic inclusion and exclusion criteria, International Classification of Diseases 9^th^ edition (ICD-9) diagnostic codes, Current Procedural Terminology (CPT) codes, and keyword exclusions from specific notes and reports of a participant in order to categorize cases and controls. Keyword searches of EMRs were conducted using natural language processing (NLP) algorithms available for those allowing EMR data for research. For individuals who lacked a diagnostic code for fibroids, but potentially had a history of fibroids documented, the NLP approach reliably identified these participants.

Eligible participants were women at least 18 years of age and required to have mention of at least one imaging procedure performed in which a fibroid would be identified were it present. Participants with procedure codes (one or more for cases and two or more on separate dates for controls) for imaging with ultrasound, magnetic resonance imaging (MRI), or computed tomography (CT) were included. Then, cases required evidence of a fibroid diagnosis defined by either an ICD-9 code indicating the presence of fibroids or ICD and CPT codes indicating a history of fibroid treatment procedures (e.g., myomectomy, uterine artery embolization, hysterectomy). Controls were participants who had two or more imaging events on separate dates without fibroids noted and did not have a fibroid diagnosis or history of fibroid treatment procedures. Women without an intact uterus (e.g., having had a prior hysterectomy) based on CPT procedural codes and text mentions of hysterectomy were considered ineligible to be controls. Controls were density matched to cases for imaging technology and timing of imaging technology within 3–5 years of case pelvic imaging to ensure comparable distribution of imaging technology across cases and controls. Finally, to reduce the potential for misclassification of would-be cases as controls, the oldest available controls were chosen within similar bins of pelvic imaging technology windows as for cases. The same algorithm was applied uniformly across all contributing eMERGE sites (Feingold-Link et al., 2014).

### Fibroid Diagnosis for UKB

Women 18 years of age or older with diagnosis of uterine leiomyomata as defined by the presence of non-cancer illness codes f.20002.0.0-28, f.20002.1.0-28, f.20002.2.0-28, or ICD10 codes f.41201.0.0-31, f.41202.0.0-379, f.41203.0.0-27, f.41204.0.0-27, and f.41205.0.0-27 were defined as fibroid cases in the UKB. All other women, over the age of 45 and for who medical codes for myomectomy, hysterectomy or uterine artery ablation were not present were considered controls. Information regarding age, and BMI at enrollment and race were abstracted.

### Genotyping and Quality Control

#### eMERGE Studies

Identical quality control (QC) was conducted within each cohort. QC summaries for each individual cohort are provided in Supplementary Table 1. QC involved excluding individuals with low genotyping efficiency (<98%), related individuals (keeping one individual from a related pair), and subjects with inconsistent reported versus genetically determined sex (Verma et al., 2014). All SNPs were tested for deviation from Hardy-Weinberg equilibrium (HWE), stratified by race (Purcell et al., 2007). We excluded SNPs with HWE *P* ≤ 10^-6^, low genotyping efficiency (<98%), minor allele frequency (MAF) < 1%, and SNPs that did not map to a chromosomal position. Furthermore, we aligned SNPs to strand by comparing allele frequencies of SNPs in each race and genotyping platform-specific GWAS set to respective allele frequencies from SNPs in the 1000 Genomes build 37 reference populations. Plots of allele frequencies were created to identify and exclude SNPs within each dataset with discordant allele frequencies (absolute value difference > 0.1) compared with their respective reference populations. Palindromic SNPs (AT and GC pairs) with MAF > 40% for which the reference strand could not be determined with certainty were also excluded.

#### UKB Analysis Sets

UKB genetic data followed standard quality control procedures. Detailed information regarding quality control and imputation can be found through the UKB documentation ^1^. Briefly, UKB samples were genotyped with the Affymetrix UK Biobank Axiom array and imputed with the Haplotype Reference Consortium (HRC) and the UK10K haplotype reference resources to yield 96 million imputed variants in 487,409 individuals (version 3; Project # 13869; accessed on Jul 31, 2018). We used these genotyped and imputed variants in women of European and African ancestry for analysis.

We used EIGENSTRAT to calculate the top 10 principal components (PCs) (Price et al., 2006). Up to five top PCs were used as covariates in regression models to control for potential confounding by population stratification.

### Statistical Analyses

IMPUTE version 2.2.2 software was used to impute ungenotyped SNPs in samples and genotypes that passed QC for the eMERGE network studies (Howie et al., 2009). GWAS data were phased using SHAPEIT software prior to imputation (Delaneau et al., 2011). The most up-to-date data from the 1000 Genomes reference panel was used as reference data to impute un-genotyped SNPs using the entire cosmopolitan panel rather than only using race/ethnicity-specific reference populations (build 37, 2013) (Howie et al., 2009). Studies have shown that using the entire reference panel increases imputation accuracy (Howie et al., 2011).

Relevant participant characteristics between fibroid cases and controls were compared with Student’s t-test with the assumption of unequal variance for continuous traits (Table 1). Stata, version 11 (StataCorp, College Station, TX, United States) was used to compare means.

We followed a two-stage genome-wide meta-analysis approach to evaluate the association between genetic markers and fibroids. Studies in Stage I were evaluated for eligible and available genotyped/imputed variants throughout the genome. Summary statistics from individual studies were meta-analyzed using inverse-variance weighted fixed effects meta-analysis approach. Selected variants (*N* = 860 SNPs) from Stage I with *P* < 1 × 10^-5^ were carried forward for investigation in Stage II. Effect estimates from Stage I and Stage II were then meta-analyzed to obtain final estimates.

The associations between each genotyped/imputed genetic marker (additive genotype model) and fibroid status were assessed using multiple logistic regression while adjusting for up to five top PCs with SNPTEST software (Marchini et al., 2007). Interpretation of analyses from logistic regression models were limited to genotyped and imputed markers which had a post-imputation information score of ≥ 0.4, HWE *P* > 1 × 10^-8^, and MAF ≥ 1%. Choice of MAF threshold was set so that the expected count of minor alleles in cases, computed with HWE assumptions, was ≥ 20 within each data set to mitigate test statistic inflation due to sparse cells for lower frequency variants (Supplementary Table 1). Associations across Stage I, Stage II and Stage I + Stage II combined analyses were performed using METAL software (Willer et al., 2010). Quantile-quantile (QQ) plots for single SNP association analyses and lambda estimates for individual studies in the discovery analysis (Supplementary Figures 1a–k and Supplementary Table 1) and their meta-analyses (Supplementary Figure 2) suggested appropriate control of genomic inflation.

Genetic variants were considered to be statistically significant and relevant for reporting if they met the following criteria: Stage I and Stage II combined *P* < 5 × 10^-8^, Stage II *P* < 0.05 and consistent directions of effect between Stage I and Stage II. At its simplest form, when there was just one GWAS significant SNP, a locus was defined as region centered around ± 500 kb of the SNP. If more than one GWAS significant SNP was present proximally in a given region, the locus for this region started 500 kb upstream from the first GWAS significant SNP and ending 500 kb downstream from the last GWAS significant SNP.

#### Conditional Analyses

We used two parallel approaches with the Genome-wide Complex Traits Analysis (GCTA) software to perform conditional analysis of common variants from the discovery GWAS summary statistics: (i) locus-specific conditional analysis, and (ii) genome-wide joint conditional analysis (Yang et al., 2011,Wu et al., 2007). We used the BioVU EA genetic data (*N* = 19,726) as the reference genotype-level data for LD approximation. Locus-specific conditional analyses were performed for SNPs with MAF > 1% in all GWAS significant loci in final meta-analysis. SNPs were considered to be conditionally significant from these analyses if the SNP remained GWAS significant after conditioning on the sentinel variant. Genome-wide joint conditional analysis was also performed using the full discovery GWAS summary statistics using the –cojo method which performs iterative conditional and joint analysis simultaneously with stepwise model selection. A *p*-value cut-off of 5 × 10^-8^ was used as the selection threshold within GCTA, and the collinearity threshold was set at the default *R*^2^ value of 0.9, so that highly correlated SNPs are not selected in the model. We then compared SNPs from both approaches and for robustness, a secondary signal was only claimed if a given SNP was validated from both approaches.

### Post-GWAS Analyses

#### RegulomeDB

We further evaluated evidence for regulatory function in SNPs identified in the genetic analyses by using RegulomeDB (Boyle et al., 2012) and the Genotype-Tissue Expression (GTEx) portal resource. RegulomeDB is a publicly available database which annotates SNPs with known and predicted regulatory elements using various sources of information including GEO, ENCODE and other published literature. The web-based tool provides a score for each SNP, where a lower score provides the greatest evidence for regulatory functionality (Boyle et al., 2012).

#### S-PrediXcan

We evaluated the association between GPGE and uterine fibroid risk using the S-PrediXcan software (Barbeira et al., 2018), a meta-analysis extension of the PrediXcan method. PrediXcan is a gene-based data aggregation and integration method which incorporates information from gene expression and GWAS data. Evidence of association with a phenotype is thus translated from the SNP-level to gene expression level (Gamazon et al., 2015). Briefly, PrediXcan first imputes gene-expression at an individual level using prediction models trained on datasets with data available on genetic variation and transcriptome expression (The GTEx Consortium et al., 2015). It then regresses the phenotype onto imputed transcriptome levels. S-PrediXcan extends its application to allow inference of the direction and magnitude of GPGE-phenotype associations with summary statistics from GWAS, which is advantageous when SNP-phenotype associations result from a meta-analysis setting and also when individual level data are not available. S-PrediXcan analyses were conducted in all 42 available GTEx tissues with modes deposited at predictdb.org, excluding the prostate and the testis. All Ps presented are two-sided.

#### eQTL-Based Summary Mendelian Randomization (SMR)

We performed two-sample eQTL based SMR analyses using GWAS significant SNPs from all 11 loci from the final meta-analysis with the SMR software (Zhu et al., 2016). Briefly, two-sample SMR is an instrumental variable approach to investigating the relationship between an exposure and outcome, in our case, gene expression in the uterine tissue and uterine fibroids. This method is particularly advantageous when exposure and outcome are not simultaneously collected. In a two-sample SMR, the association between gene expression and outcome is approximated taking a ratio of the association between SNP and outcome (GWAS summary statistics) and SNP and gene expression (eQTL of desired tissue from GTEx), β_SMR(Gene-Expression-fibroids)_ = β_SNP-fibroids_/ β_SNP-eQTL-GTEx uterine tissue_.

As input for the SMR analysis we provided summary statistics for eQTLs in the GTEx uterine tissue, summary statistics for GWAS significant SNPs at each locus from GWAS analysis, and the 1000-Genomes reference population for LD estimation. Analyses were performed separately at each locus allowing inclusion of GWAS significant SNPs with *R*^2^ < 0.9, and overlapping eQTL SNPs with *P*-values < 0.05. A *cis*-eQTL-gene pair was not specified, rather, the software was allowed to evaluate and report associations for *cis*-eQTL and gene pairs in a region contained within ± 2000 KB of the index GWAS significant SNP. Data are automatically harmonized to choose consistent effect allele between summary GWAS and summary eQTL for calculation of beta-estimates. The output allows inference of association between gene expression at each locus and uterine fibroids. When more than one eQTL-gene pair was identified (GWAS significant SNP and eQTL *P*-value < 0.05) for a given locus, these SMR results were sorted by *P*-value and reported.

#### DEPICT

Enrichment analyses in DEPICT (Pers et al., 2015) were conducted using SNPs less than two *P*-value thresholds: *P* < 5 × 10^-7^, and *P* < 1 × 10^-5^. DEPICT is based on predefined phenotypic gene sets from multiple databases and Affymetrix HGU133a2.0 expression microarray data from more than > 37k subjects to build highly expressed gene sets for Medical Subject Heading (MeSH) tissue and cell type annotations. Output includes a *P* for enrichment and a yes/no indicator of whether the FDR *q*-value is < 0.05. Tissue level and gene-set enrichment features are considered.

## Results

We summarized participant characteristics in our study populations in Table 1. Four of the nine studies in the imaging-confirmed electronic medical record (EMR) case-control sets from the eMERGE network were identified as AA, with the remaining comprised of EA individuals. Participants from the UKB were classified as African or European. Uterine fibroid cases were younger than fibroid controls (*P* < 0.05) in eight of the thirteen studies (Table 1). In agreement with previously published studies, fibroid cases had higher mean BMI than controls, with significant differences in six of the thirteen studies (Table 1).

### Single Variant Analyses

**FIGURE 2:**
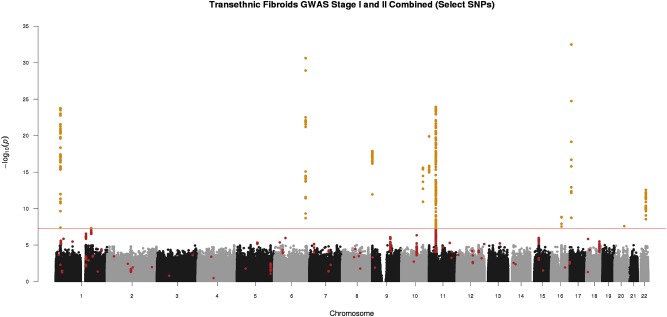
Manhattan plot representing Stage I and II *trans*-ethnic meta-analyses. Black and gray dots represent Stage I SNPs only; red dots represent SNPs with suggestive or greater evidence (*P* < 1 × 10^-5^) in Stage I, but not replicated in Stage II (*P* > 0.05). Golden dots represent SNPs with *P* < 5 × 10^-8^ after meta-analysis of Stage I and Stage II + Stage II *P*-value < 0.05 + consistent directions of effect between Stage I and Stage II

We conducted a two-stage analysis, discovery GWAS and targeted replication, to investigate the association between single variants and fibroids (Figure 1). Genome-wide discovery meta-analysis in 9,446 cases and 67,048 controls from 11 ancestry-specific datasets from eMERGE and UKB (Stage I) identified 124 significant GWAS SNPs (*P* < 5 × 10^-8^), and 860 variants with suggestive evidence (*P* < 1 × 10^-5^). Variants with significant and suggestive evidence were tested for replication in UKB (Stage II) African and European ancestry subsets (Stage II *N* = 12,358 cases, 138,477 controls). Meta-analysis of Stage I and Stage II effect estimates identified 326 variants from 11 loci that surpassed GWAS significance, had a *P* < 0.05 in Stage II replication and had consistent directions of effect between discovery and replication meta-analysis sets (Figure 2, Table 2, and Supplementary Figures 2–13).

**Table 2:** Sentinel SNPs from *trans*-ethnic genome-wide meta-analysis of 13 African and European ancestry studies.

SNP	CHR	BP	EFF/ OTH	Nearby Genes	Freq	OR	(95% CI)	*P*	Direction	HetP
rs17361789#	1	172,122,601	T/G	*DNM3^∗^*	0.68	0.94	(0.92, 0.96)	4.67 × 10^-8^	+—??–+?–+	0.29
rs4785384#	16	50,147,993	T/C	*HEATR3*	0.73	1.08	(1.05, 1.10)	1.45 × 10^-9^	++++-++++++++	0.38
rs6094982#	20	46,761,257	A/T	*LOC105372640*	0.04	2.09	(1.61, 2.70)	2.55 × 10^-8^	+??+???????++	0.05
rs10917151	1	22,422,721	A/G	*CDC42, WNT4*	0.16	1.16	(1.13, 1.19)	1.76 × 10^-24^	-++-??+++?++-	0.41
rs58415480	6	152,562,271	C/G	*SYNE1^∗^*	0.84	0.84	(0.82, 0.87)	2.39 × 10^-31^	——–+—-	0.73
rs1812264	9	805,427	T/G	*LOC105375949^∗^*	0.61	0.91	(0.89, 0.93)	1.31 × 10^-18^	+++-+–++–-	0.15
rs7907606	10	105,680,632	T/G	*OBFC1*	0.81	0.89	(0.87, 0.92)	2.49 × 10^-16^	—-+–+–-	0.81
rs7124615	11	186,604	T/C	*SCGB1C1, BET1L*	0.14	0.86	(0.83, 0.89)	1.28 × 10^-20^	+–+++–+–-	0.12
rs10835889	11	32,370,380	A/G	*WT1*	0.18	1.16	(1.13, 1.19)	1.17 × 10^-24^	+++++++++++++	0.96
rs78378222	17	7,571,752	T/G	*TP53^∗∗^*	0.99	0.54	(0.49, 0.60)	3.24 × 10^-33^	?-+???????–?	0.11
rs3830738	22	40,711,227	A/AT	*TNRC6B^∗^*	0.79	0.91	(0.89, 0.93)	2.73 × 10^-13^	-+–+—++—	0.21


*SNP, Single Nucleotide Polymorphism; CHR, Chromosome; BP, SNP Base Position in dbSNP build 37; EFF, Effect allele; OTH, other allele; Freq, frequency for effect allele averaged across all relevant studies; OR, odds ratio; 95% CI, 95% Confidence Interval; Het-P, P for test assessing heterogeneity across studies. Order of studies for the direction column: BioVU AA, BioVU EA, BioVU Stage II EA, BioME AA, Mount Sinai AA, eMERGE I AA, eMERGE I EA, Northwestern EA, Mayo EA, UKB AA (Stage I), UKB EA (Stage I); UKB EA (Stage II), UKB AA (Stage II) #Novel loci; ^∗^ = Intron variant; ^∗∗^ = 5′/3′ UTR variant.*

**FIGURE 3:**
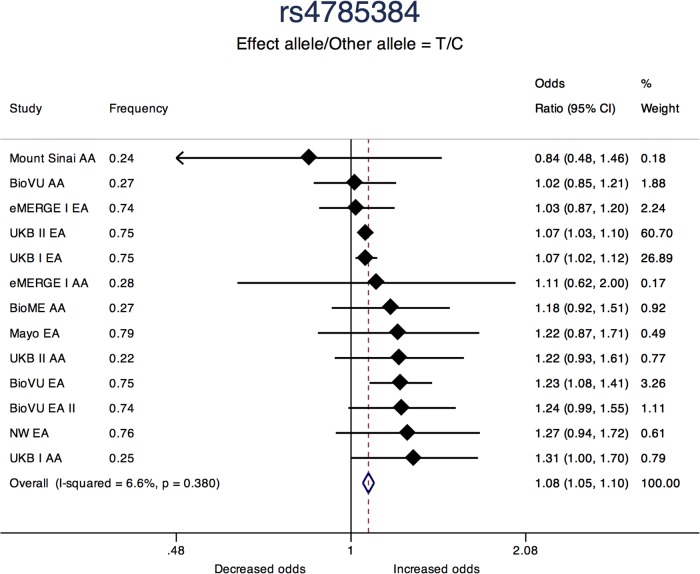
Forest plot showing odds ratio estimates from individual studies and meta-analysis for SNP rs4785384. Meta-analyses were conducted using inverse-variance weighted fixed-effects meta-analysis to present odds ratios and 95% confidence intervals. Study column represents participating study names by strata of race. FREQ represents allele frequency for the effect allele coded in the plot.

Three of the 11 loci are novel. Among novel loci, sentinel SNP rs4785384, on chromosome 16q12.1 near HEAT repeat containing 3 (*HEATR3*) was most significantly associated with fibroids (T allele Odds Ratio [OR] = 1.08; 95% confidence interval [CI]: 1.05, 1.10; *P* = 1.45 × 10^-9^; heterogeneity p [P-Het] = 0.38). The SNP had consistent directions of effect in 12 out of 13 studies (Figure 3). Sentinel SNP rs78378222, located in the 3′ untranslated region of the tumor protein p53 (*TP53*) gene, a known fibroid locus, was the most significant association in the study (T allele OR = 0.54; 95% CI: 0.49, 0.60; *P* = 3.24 × 10^-33^; Het-P = 0.11). This variant is monomorphic in the African reference populations and was an uncommon allele only observed in datasets with European ancestry individuals (minor allele frequency [MAF] = 1%).

**FIGURE 4:**
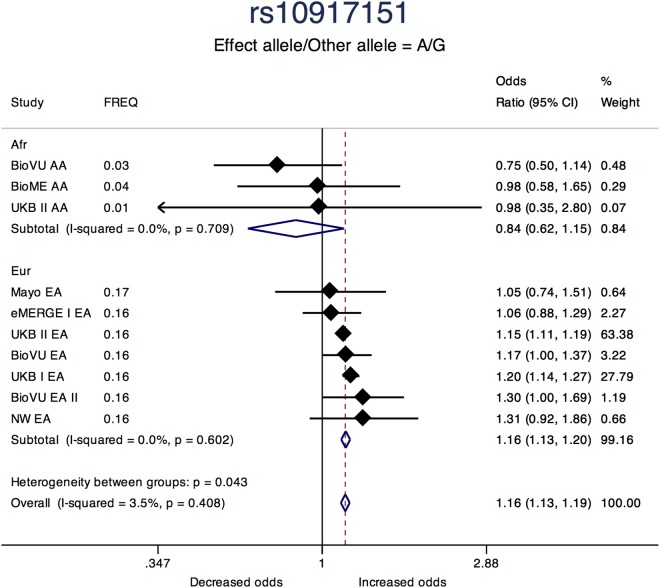
Forest plot showing odds ratio estimates from individual studies and meta-analysis for SNP rs10917151 by continental ancestry strata – African and European origin. Meta-analyses were conducted using inverse-variance weighted fixed-effects meta-analysis to present odds ratios and 95% confidence intervals for studies within each continental ancestry strata. Study column represents participating study names by strata of race. FREQ represents allele frequency for the effect allele coded in the plot. Heterogeneity between groups P compares odds ratios between the two strata.

Sentinel SNPs from other notable loci previously reported in the literature include rs58415480 (*P* = 2.39 × 10^-31^), an intron variant in the spectrin repeat containing nuclear envelope protein 1 (*SYNE1*) gene, rs10835889 (*P* = 1.17 × 10^-24^) near the Wilms Tumor 1 (*WT1*) gene, and rs3830738 (*P* = 2.73 × 10^-13^), an intron variant in the trinucleotide repeat containing 6B (*TNRC6B*) gene (Table 2). Directions of effect were consistent across ancestry groups (African and European) for these associations. SNP rs10917151 (*P* = 1.76 × 10^-24^) located between cell division cycle 42 (*CDC42*) and the Wnt family member 4 (*WNT4*) genes was positively associated with uterine fibroids (A allele OR = 1.16; 95% CI: 1.13, 1.19). However, stratifying analyses by race (African and European) showed effect estimates for African and European ancestry were in opposite directions (inverse, statistically non-significant association for African (OR = 0.84; 95% CI: 0.62, 1.15) and positive association for European ancestry (OR = 1.16; 95%: 1.13, 1.20); Het-P-between-groups = 0.04; Figure 4).

Locus-specific conditional analyses of the discovery GWAS summary statistics with GCTA did not identify additional secondary signals at any of the 11 loci after conditioning on the sentinel SNP at each locus. Genome-wide joint conditional analysis of discovery GWAS data identified five variants that remained significant before and after joint-conditional analyses (Supplementary Table 2). These jointly modeled SNPs are independent of each other, but not after conditioning on the sentinel variant. Two of these five SNPs were the sentinel SNPs at the locus (Supplementary Table 2).

### Regulatory Annotations

We utilized the RegulomeDB resource to investigate the presence of genetic variants with regulatory effects among GWAS significant variants recognized by RegulomeDB (308 out of 326 SNPs) (Boyle et al., 2012). RegulomeDB scores aggregate evidence for the presence of regulatory motifs for SNPs which are ranked from 1a (most evidence) to 7 (no data). One hundred thirty-eight variants were scored as regulatory (score of ≤ 5) (Supplementary Table 3). One SNP in chromosome 16q12.1, rs6500288 (score: 1f), showed the greatest evidence for being in a regulatory region, suggesting the SNP is an eQTL for a nearby gene. The GTEx Project portal showed this SNP is a significant eQTL for the *HEATR3* gene in the lung, tibial nerve, thyroid, tibial artery, esophagus muscularis, and several other tissues (Largest *P* = 1 × 10^-6^ in the liver; and Smallest *P* = 1.9 × 10^-50^ in the lung). Six SNPs on chromosome 11, four SNPs on chromosome 1, two SNPs on chromosome 2, two SNPs on chromosome 9 and one SNP on chromosome 17 also were scored as regulatory with RegulomeDB scores of 2a and 2b.

### Transcriptome-Wide Association Analysis

**FIGURE 5:**
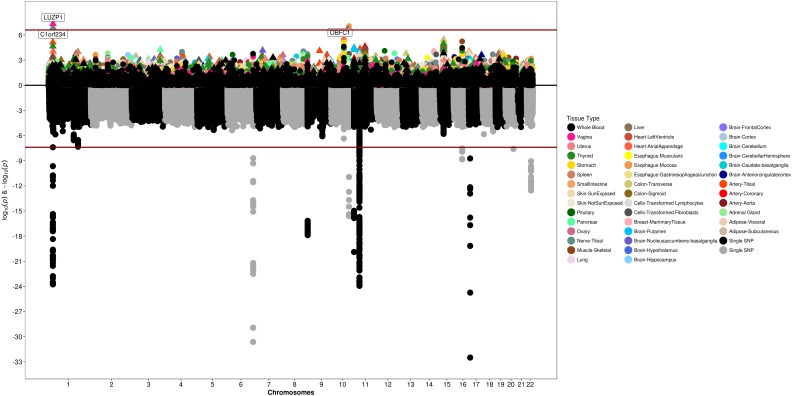
Genetically predicted gene expression in 42 GTEx tissues and uterine fibroids with S-PrediXcan. Top panel represents gene-level –log10 *P*-values from S-PrediXcan; bottom panel dots represent log10 *P*-values from GWAS evaluating SNPs and uterine fibroids.

**FIGURE 6:**
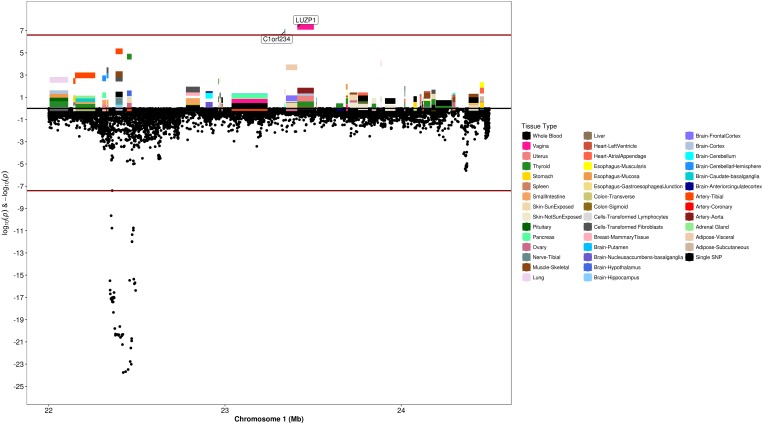
Regional S-PrediXcan and SNP association plot for *WNT4, CDC42*, and *LUZP1* loci. **(Top)** Represents gene-level –log10 *P*-values from S-PrediXcan; **(bottom)** dots represent log10 *P*-values from GWAS evaluating SNPs and uterine fibroids.

**FIGURE 7:**
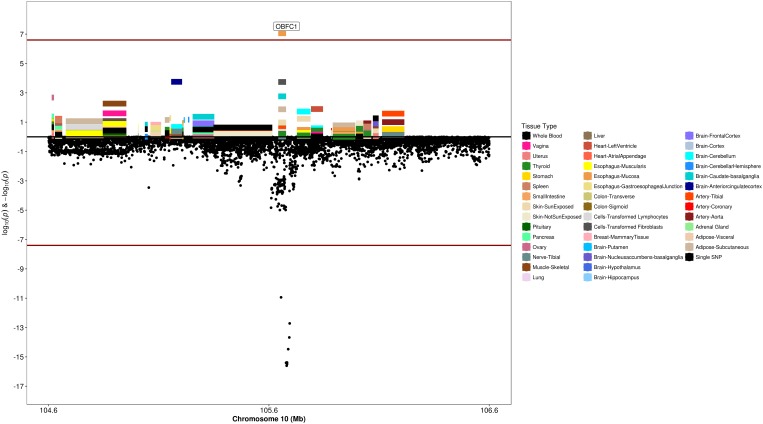
Regional S-PrediXcan and SNP association plot for *OBFC1* locus. **(Top)** Represents gene-level –log10 *P*-values from S-PrediXcan; **(bottom)** dots represent log10 *P*-values from GWAS evaluating SNPs and uterine fibroids.

We used S-PrediXcan along with 42 tissues from the GTEx project (excluding male-specific tissues) to identify associations between GPGE and uterine fibroid risk (Figure 5 and Table 3). Increasing GPGE of *LUZP1* in the vagina tissue was positively associated with uterine fibroids (*Z*-score = 5.47; *P* = 4.6 × 10^-8^; Figure 6 and Table 3). The gene lies in the same chromosome 1p36.12 region as the *CDC42* and *WNT4* genes. There was suggestive evidence for predicted expression of *CDC42* in the tibial artery and fibroids (*Z*-score = 4.48; *P* = 7.4 × 10^-6^). GPGE analyses also identify *OBFC1* on chromosome 10. *OBFC1* expression in the esophagus mucosa was inversely associated with uterine fibroids (*Z*-score = -5.35; *P* = 8.7 × 10^-8^; Figure 7 and Table 3). Additionally, increased GPGE of *NUDT13* across multiple tissues (subcutaneous adipose transformed lymphoblasts, esophagus muscularis, and breast mammary tissue) was positively associated with fibroid risk (Table 3). Consistent with the GWAS nearest-gene approach, RegulomeDB prediction and GTEx eQTL confirmation, increasing GPGE of *HEATR3* in skeletal muscle was positively associated with fibroids (*P* = 5.8 × 10^-6^). Although we did not find a significant association between GPGE of *WT1* and fibroids, its RNA expression is highest in the uterus tissue (N samples = 111), followed by the fallopian tube (N samples = 7), and ovary (N samples = 133) (Figure 7). The GTEx portal does not identify strong eQTLs for the *WT1* gene.

**Table 3:** Summary of genetically predicted gene expression and fibroids risk in 42 GTEx tissues.

Gene	Region	*Z*-Score	*P*	Var-G	*R*^2^	*Q*-value	SNPs Used N	SNPs in Model N	Tissue
*LUZP1*	1p36.12	5.47	4.6 × 10^-8^	0.068	0.102	1.4 × 10^-2^	13	13	Vagina
*OBFC1*	10q24.33	-5.35	8.7 × 10^-8^	0.062	0.050	3.9 × 10^-4^	33	38	Esophagus Mucosa
*C1orf234*	1p36.12	-5.28	1.3 × 10^-7^	0.009	0.010	4.0 × 10^-2^	5	5	Tibial Nerve
*NUDT13*	10q22.2	4.65	3.3 × 10^-6^	0.300	0.292	3.0 × 10^-23^	100	102	Subcutaneous Adipose
*MAPKBP1*	15q15.1	-4.64	3.5 × 10^-6^	0.020	0.038	4.8 × 10^-4^	17	19	Subcutaneous Adipose
*DNAJC9*	10q22.2	4.63	3.7 × 10^-6^	0.052	0.059	2.8 × 10^-3^	55	56	Atrial Appendage
*NUDT13*	10q22.2	4.57	4.9 × 10^-6^	0.271	0.299	2.7 × 10^-9^	51	55	Transformed Lymphoblasts
*NUDT13*	10q22.2	4.56	5.2 × 10^-6^	0.317	0.379	3.2 × 10^-23^	114	118	Esophagus Muscularis
*HEATR3*	16q12.1	4.53	5.8 × 10^-6^	0.128	0.169	1.1 × 10^-15^	48	49	Skeletal Muscle
*CDC42*	1p36.12	4.48	7.4 × 10^-6^	0.012	0.008	4.4 × 10^-2^	22	22	Tibial Artery
*FAM149B1*	10q22.2	-4.48	7.4 × 10^-6^	0.014	0.038	1.2 × 10^-2^	33	36	Stomach
*PLA2G4B*	15q15.1	4.48	7.6 × 10^-6^	0.044	0.065	1.3 × 10^-3^	12	12	Stomach
*JMJD7*	15q15.1	4.47	7.7 × 10^-6^	0.196	0.362	3.0 × 10^-30^	26	28	Sun Exposed Skin Lower Leg
*NUDT13*	10q22.2	4.47	7.9 × 10^-6^	0.181	0.303	7.2 × 10^-15^	112	113	Breast Mammary Tissue
*NUDT13*	10q22.2	4.43	9.2 × 10^-6^	0.343	0.368	2.2 × 10^-26^	75	77	Tibial Nerve
*PLA2G4B*	15q15.1	4.43	9.6 × 10^-6^	0.022	0.082	1.6 × 10^-4^	9	10	Visceral Omentum Adipose


*Var-G, variance of the gene’s predicted expression, calculated as *W*’ ×*G* × *W* (where *W* is the vector of SNP weights in a gene’s model, *W*’ is its transpose, and *G* is the covariance matrix); R^2^, Performance prediction correlation; *Q*-value, FDR derived *Q*-value. Only predicted gene models with FDR *Q*-values < 0.05 are considered for genetically predicted gene expression association; SNPs used N, number of SNPs included in the prediction model for that gene available in the summary statistics; SNPs in Model N, number of SNPs used to construct the prediction model for the gene in the tissue of interest using the GTEx data; Bonferroni Significance (Sum of N-genes across all tissues tested): 0.05/194549 = 2.57 × 10^-7;^Suggestive Significance (N-Unique genes tested across all tissues tested): 0.05/17433 = 2.87 × 10^-6^.*

### Summary Mendelian Randomization

We identified associations between gene expression in the uterine tissue and fibroids in eight of the eleven GWAS significant loci investigated with SMR (Supplementary Table 4). *HEATR3*, located in 16q12.1 was the most significant association in SMR analyses, and its gene expression was positively associated with uterine fibroids (β_SMR_= 0.165; *P*-value 1.1 × 10^-5^). SMR for this gene was evaluated using rs6500288, a SNP that is in perfect LD (*R*^2^ = 1) with the index SNP rs4785384. *WT1-AS* was the most significant gene in the 11p13 locus and was positively associated with uterine fibroids (β_SMR_ = 0.22; *P*-value = 2.2 × 10^-3^). The 1p36.12 locus harboring *WNT4* showed evidence for several candidate genes including *ECE1* (β_SMR_= 0.33; *P*-value = 4.9 × 10^-3^). *WNT4* was positively associated with uterine fibroids, however, the statistical evidence was marginal (β_SMR_ = 0.57; *P*-value = 5.57 × 10^-2^).

### Tissue Enrichment Analyses With DEPICT

We further applied the DEPICT method to the top results from our GWAS analyses (*P* < 5 × 10^-7^) to evaluate evidence of tissue or gene-pathway level enrichment. Although results were not statistically significant after considering multiple comparisons (FDR < 5%), the top enriched tissue from these analyses was myometrium as the first-level MeSH term and the urogenital system as corresponding top second-level MeSH term (*P* = 0.056) (Supplementary Table 5). The urogenital system accounted for eight out of the top 20 MeSH groupings based GWAS results from this study. Similar results were obtained when the DEPICT method was applied with SNPs with *P* < 1 × 10^-5^ (Supplementary Table 6).

## Discussion

Several lines of evidence support that there is a genetic component of uterine fibroid risk. However, because of study design and ascertainment challenges, there have been few large GWAS for fibroids. Only one study, previously published by our research group, has included AAs with fibroid cases and controls verified by imaging, and only one recent study has meta-analyzed multiple ancestral populations with relatively small number of women with African ancestry. Here we investigated common genetic variants and risk for fibroids in AA and EA women with image-verified classification of case-control status from EMRs in the US and in women of European and African ancestry with diagnosis-verified fibroid case status from the UKB. We report associations for 11 genome-wide significant loci, of which eight have been previously reported and three are novel. We add evidence to relevant gene targets in relation to uterine fibroid risk by integrating GWAS data with GPGE weights across 42 tissues, and by performing locus-specific eQTL SMR analyses with the uterine tissue.

Sentinel SNP rs4785384, the most significant novel variant, located near the *HEATR3* gene had consistent directions of association in 12 out of 13 race- and site- specific assessments in this study. Increasing GPGE of *HEATR3* in skeletal muscle was positively associated with fibroids and SMR analyses suggested increasing *HEATR3* expression in the uterine tissue is positively associated with uterine fibroids. The gene product of *HEATR3* plays a role in the transport and assembly of the ribosomal 5S ribonucleoprotein particle. In recent findings, genetic variants related to *HEATR3* have been associated with increased risk of glioblastoma (Melin et al., 2017a) and esophageal cancer (Jia et al., 2015). The effect allele is common in all 1000G populations, and provides an example of a genetic association for fibroids that is shared across ethnicities.

The second novel locus, *DNM3* (sentinel-SNP: rs17361789), was proposed as a tumor suppressor gene for hepatocellular carcinoma (Inokawa et al., 2013) and is suspected to control carcinoma growth by activating p53 (Gu et al., 2017, p. 3), which is the gene product of the most significant locus detected in our study Tumor Protein 53 (*TP53*) (sentinel-SNP: rs78378222). *TP53*, a tumor suppressor gene, is one the most frequently mutated genes in many human cancers (Kastenhuber and Lowe, 2017) and has also been associated with fibroids (Rafnar et al., 2018). The sentinel variant for *TP53* is polymorphic in European derived reference populations and monomorphic in African populations in the 1000 genomes reference providing further evidence for presence of race-specific risk loci in relation to uterine fibroids.

Our study also provides evidence of association for several previously reported genetic loci including *TNRC6B, BET1L and OBFC1*. First shown by Cha et al. (2011a) in a Japanese population in 2011, variants from these genes have since been associated with uterine fibroid risk in candidate gene studies conducted in Saudi women (Bondagji et al., 2017), white women (Edwards et al., 2013b), and now in this *trans*-ethnic analysis of white and black women from the United States and the United Kingdom. Another study from our group reported associations between variations in the *TNRC6B* gene and fibroid volume (Edwards et al., 2013a).

Several studies have reported associations between variants near *OBFC1* and fibroids (Cha et al., 2011b; Rafnar et al., 2018; Välimäki et al., 2018). *OBFC1* has been suggested to play a role in maintaining telomere length and SNPs in this locus have been associated with many cancers including melanoma (Law et al., 2015; Ransohoff et al., 2017), glioma and non-glioblastoma glioma (Melin et al., 2017b), thyroid (Gudmundsson et al., 2017), basal cell carcinoma (Chahal et al., 2016), renal cell carcinoma (Scelo et al., 2017), ovarian cancer (Phelan et al., 2017), and lung adenocarcinoma (McKay et al., 2017). Our findings are the first to observe that increased GPGE of *OBFC1* is associated with reduced risk of uterine fibroids, although Phelan et al noted that the sentinel SNP in *OBFC1* for ovarian cancer was likely regulatory (Phelan et al., 2017).

The *WT1* gene lies within 100 kb of the index SNP (rs11828433) on chromosome 11p13. Index SNP rs11828433, along with other genome-wide significant variants in this region showed consistent directions of association across studies and is a common variant in European and African ancestral populations. *WT1* encodes a transcription factor and plays a crucial role in the development of the urogenital system. Among all tissues in GTEx, *WT1* is most expressed in the uterine tissue, followed by other reproductive tissues (Figure 5). Haploinsufficiency of *WT1* is associated with Wilms tumor, while dysregulated *WT1* expression is associated with tumors including leukemia, testicular germ cell tumors and uterine leiomyosarcoma (Coosemans et al., 2011; Boublikova et al., 2016; Cebinelli et al., 2016; Naitoh et al., 2016). Two other studies have reported marginal associations between variants rs12789861 (Shmygelska et al., 2013), and rs1223079 (Aissani et al., 2015) at this locus and fibroids (Supplementary Figure 14); and the most recent study showed genome-wide significant associations at this locus (Rafnar et al., 2018).

The chromosome 1p36.12 region containing the *CDC42* and *WNT4* genes, was statistically the second most significant region from GWAS analysis. *CDC42* and *WNT4* were the closest genes to the top SNPs in this locus, however, incorporating tissue expression data suggested Leucine Zipper Protein 1 (*LUZP1*) expression in the vagina, rather than *WNT4* or *CDC42*, as the gene most associated with fibroid risk. *LUZP1* has been implicated as a negative transcription regulator; however, the mechanism by which transcript abundance of this gene would affect uterine fibroid risk is unclear. Increased expression of *WNT4* has been found in studies of fibroid tumors that carry somatic *MED12* mutations (Markowski et al., 2012). However, it is not clear from somatic studies if the increase in *WNT4* expression is a consequence of uterine fibroid growth, or a cause. In contrast with the somatic studies that support a positive relationship between *WNT4* expression and the fibroid state, increasing GPGE of *WNT4* in thyroid tissue is non-significantly inversely associated with uterine fibroid risk (*P* = 2.2 × 10^-5^). A deeper evaluation of the index SNP rs10917151 in the GTEx database showed this SNP is not a strong eQTL for *WNT4* and the direction of association varies by tissue type, with a non-significant yet positive association in the uterus (Supplementary Figure 14). Our SMR analyses in region suggested a positive association between *WNT4* expression in the uterine tissue and fibroids risk, as shown in the literature. However, our results also highlight the possibility that other genes in this locus may be associated with uterine fibroids.

This study also reports evidence of association with ancestry-specific alleles that may influence fibroid risk and health disparities between populations. The top variant in the *WNT4/CDC42* had opposite directions of effect in African and European ancestry women, and associated variants in the *TP53* locus were only polymorphic in European women. We have previously reported evidence for differential burdens of risk-increasing alleles for fibroproliferative traits across global populations that are consistent with prevalence disparities (Hellwege et al., 2017b). This finding was recently extended to evaluate only fibroid risk variants across populations, with an increased mean burden of risk alleles in black fibroid cases and controls from UKB compared to other racial/ethnic groups, with a similar result using frequencies from the gnomAD browser (Välimäki et al., 2018).

This study is the largest multi-stage *trans*-ethnic genome-wide investigation of uterine fibroid risk. Utilizing validated algorithms for fibroid case-control identification in multiple EMR-linked biobanks in the United States, and diagnosis-confirmed fibroid cases with appropriate female controls in the UKB, this study verifies several previously reported loci, reports variants at novel loci, and extends inferences using summary Mendelian randomization and predicted gene expression in relation to uterine fibroids. The study also provides evidence for the presence of shared and ancestry specific genetic loci which may influence fibroid risk and disparities. Further fine-mapping, and characterization of mechanisms involved will help elucidate the role of variants and genes reported in this study.

## Ethics Statement

The Institutional Review Boards at all participating institutions in the eMERGE network approved this study. Genetic data for the eMERGE network are available through dbGaP. Researchers had access to de-identified data only, and the investigators received non-human subject determination from the Vanderbilt University Medical Center.

## Author Contributions

DE and TE conceived the work. DE, TE, ES, RG, HK, EK, CM, MW, GT, JD, DR, AK, and PP acquired the data. TE, AG, JH, JJ, MB, SP, ET, JK, KJ, JAP, JP, and PP analyzed the data. HI and ET developed the software. TE, AG, JH, and DE wrote the manuscript. TE, AG, JH, KH, ES, JJ, MB, SP, ET, JK, SJ, RG, HK, KJ, AK, IK, CM, HI, JAP, JP, MW, GT, EK, PP, JD, DR, and DE interpreted the work and critically reviewed the manuscript. DE, TE, and KH supervised the work. DE contributed to funding of the work. TE, AG, JH, KH, ES, JJ, MB, SP, ET, JK, SJ, RG, HK, KJ, AK, IK, CM, HI, JAP, JP, MW, GT, EK, PP, JD, DR, and DE approved the final version to be published.

## Conflict of Interest Statement

The authors declare that the research was conducted in the absence of any commercial or financial relationships that could be construed as a potential conflict of interest.

## Footnotes

1 www.ukbiobank.ac.uk
