# Supplementary material for: A Trans-Ethnic Genome-Wide Association Study of Uterine Fibroids
Source: Front Genet. 2019 Jun 12;10:511. doi: 10.3389/fgene.2019.00511 (PMC6582231; doi:10.3389/fgene.2019.00511)
Supplement: Supplementary file 1 [file Data_Sheet_1.PDF]

Supplementary Figures 1a-k: Quantile-Quantile plots for studies included in the discovery analysis

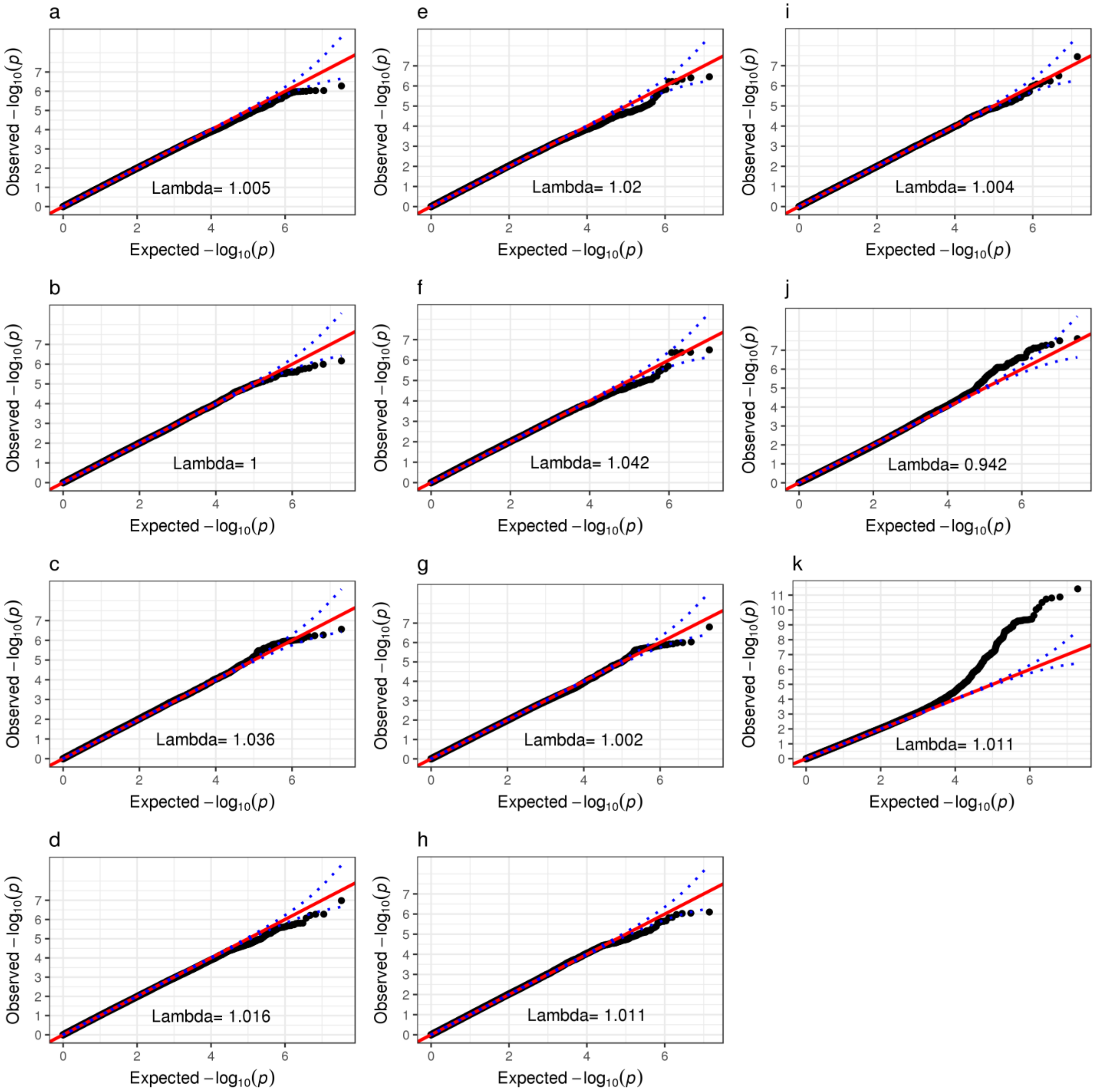

a = BioVU AA; b = BioVU EA; c = BioVU-II EA; d = Mount Sinai: BioMe AA; e = Mount Sinai AA; f = eMERGE I (Northwestern) AA; g = eMERGE I (Marshfield; Northwestern; Group Health) EA; h = Northwestern EA; i = Mayo Clinic EA; j = UK Biobank African; k = UK Biobank European; AA = African American; EA = European American

Supplementary Figure 2: Quantile-Quantile plot for discovery meta-analysis

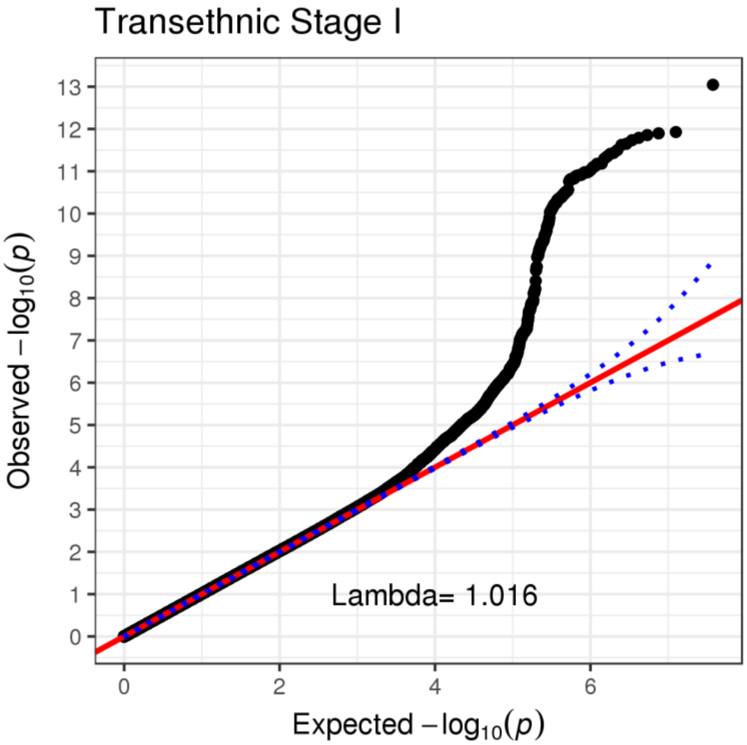

Supplementary Figures 3a-b: Regional association plots for rs17361789 from trans-ethnic final meta-analysis using the 1000 Genomes LD for African-ancestry (a) and European ancestry (b)

a

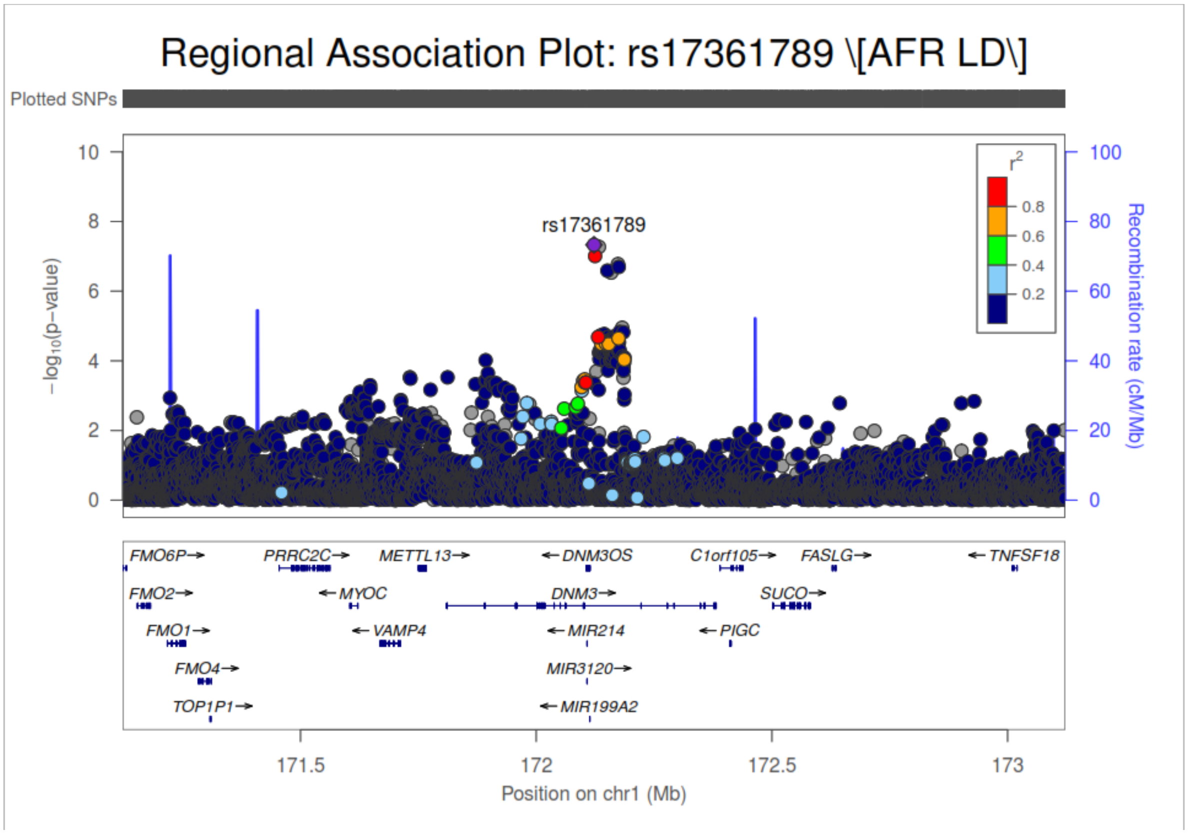

b

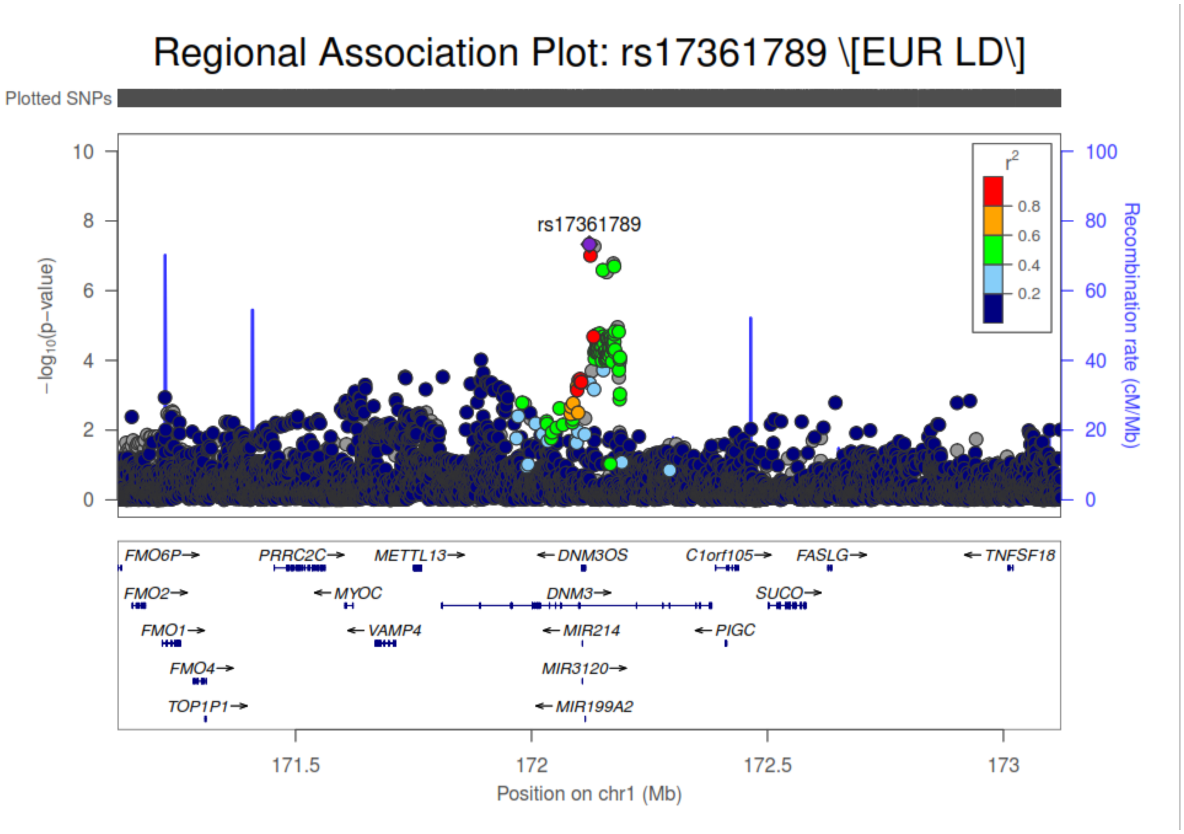

Supplementary Figures 4a-b: Regional association plots for rs4785384 from trans-ethnic final meta-analysis using the 1000 Genomes LD for African-ancestry (a) and European ancestry (b)

a

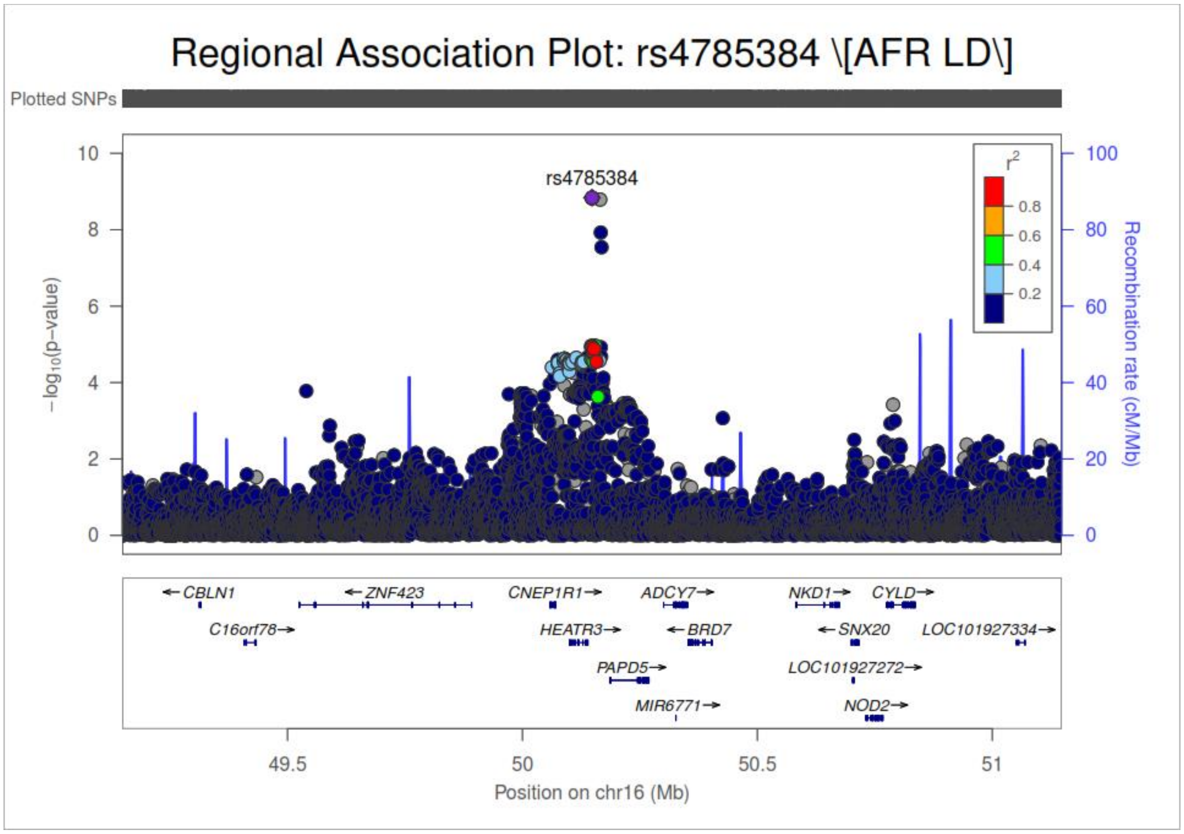

b

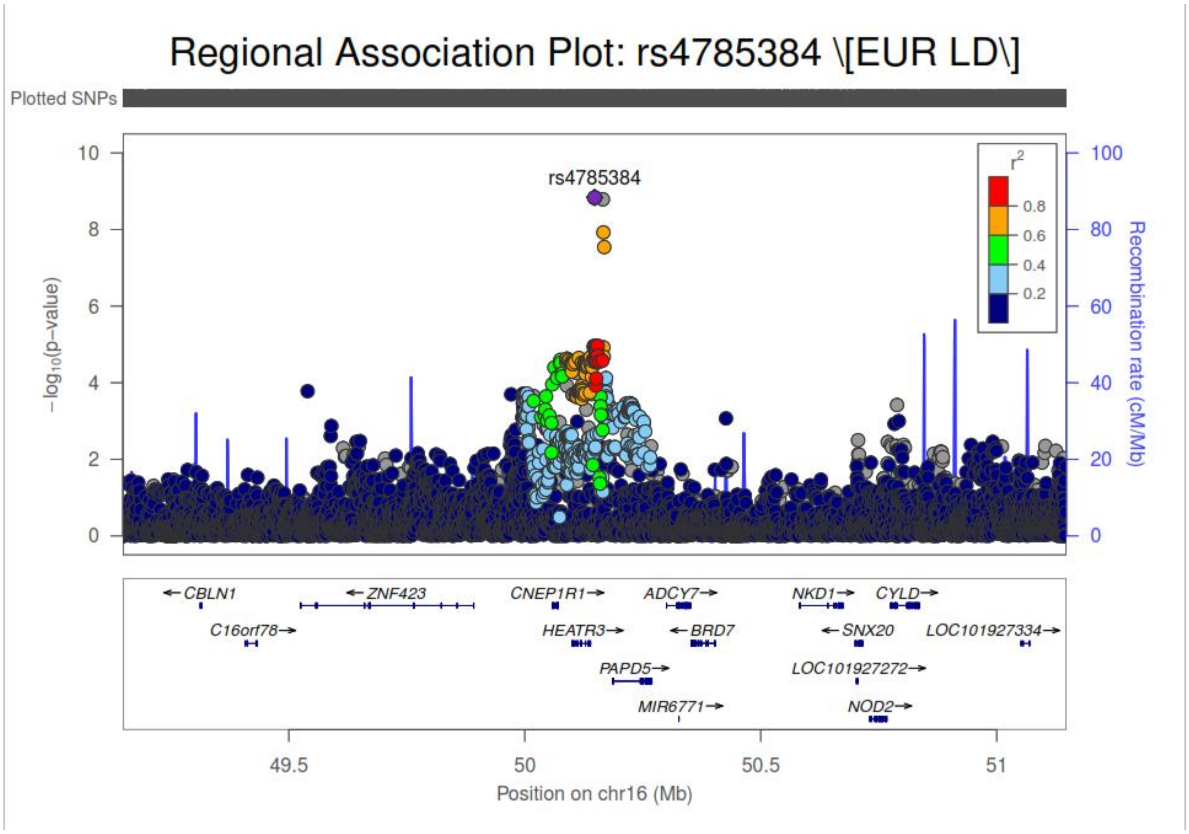

Supplementary Figures 5a-b: Regional association plots for rs6094982 from trans-ethnic final meta-analysis using the 1000 Genomes LD for African-ancestry (a) and European ancestry (b)

a

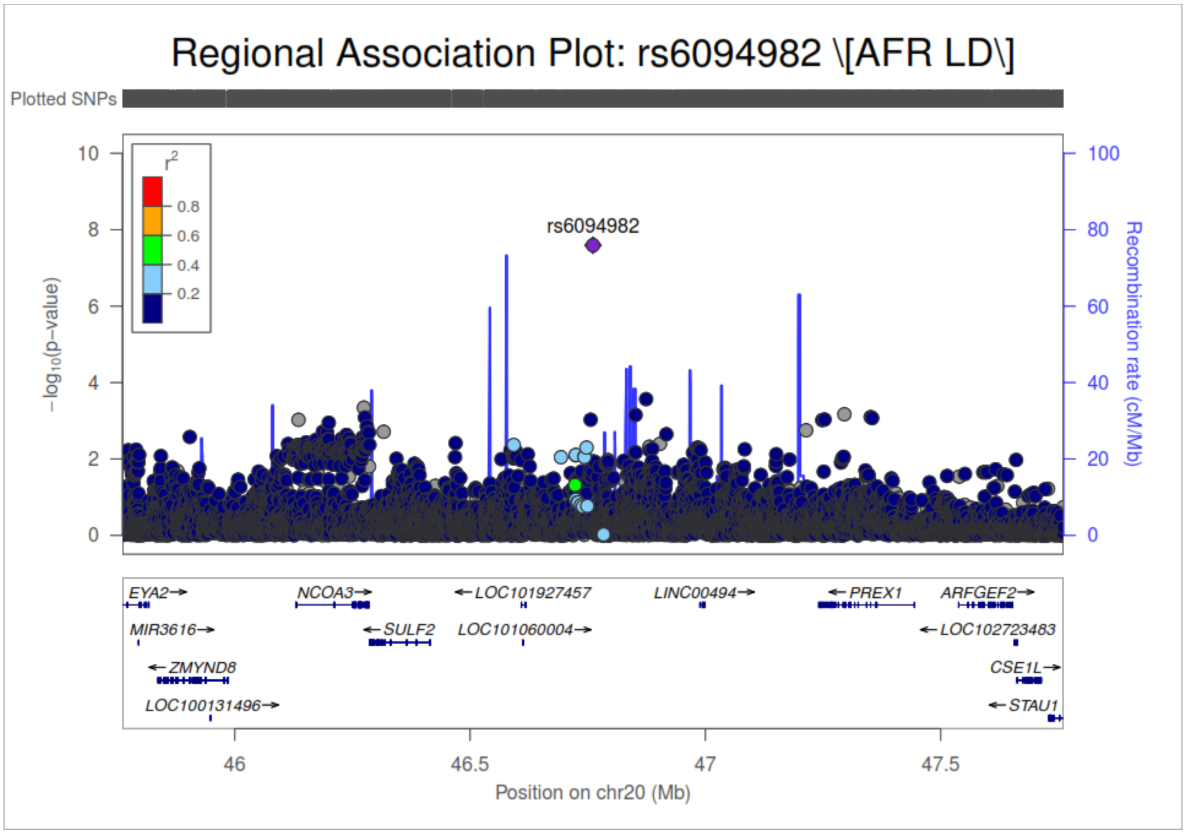

b

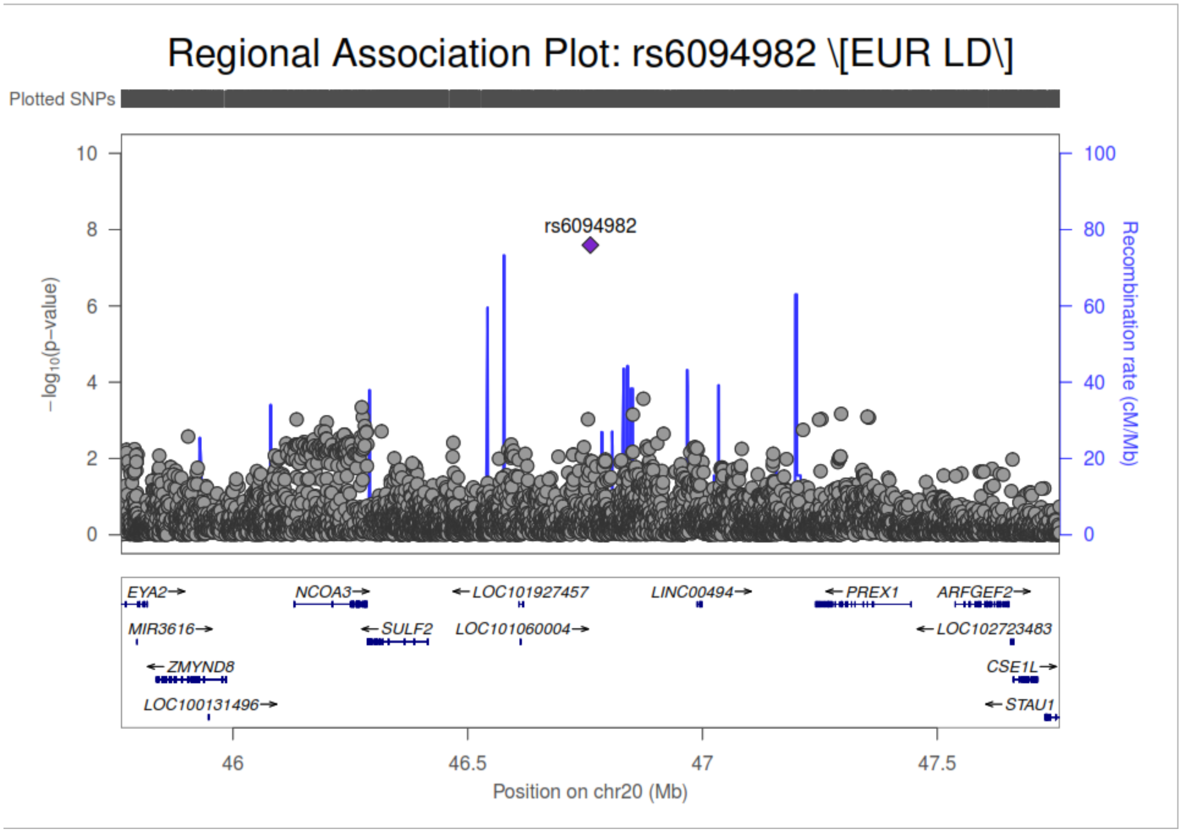

Supplementary Figures 6a-b: Regional association plots for rs10917151 from trans-ethnic final meta-analysis using the 1000 Genomes LD for African-ancestry (a) and European ancestry (b)

b

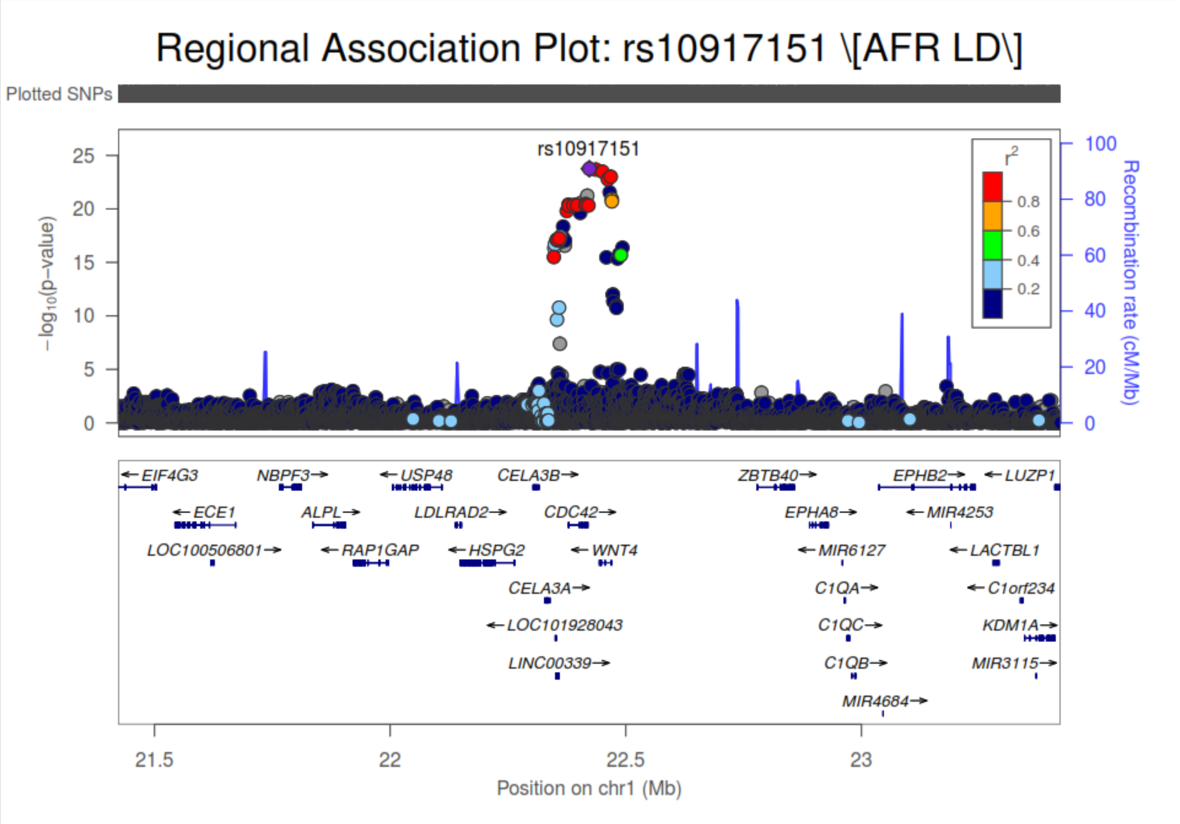

b

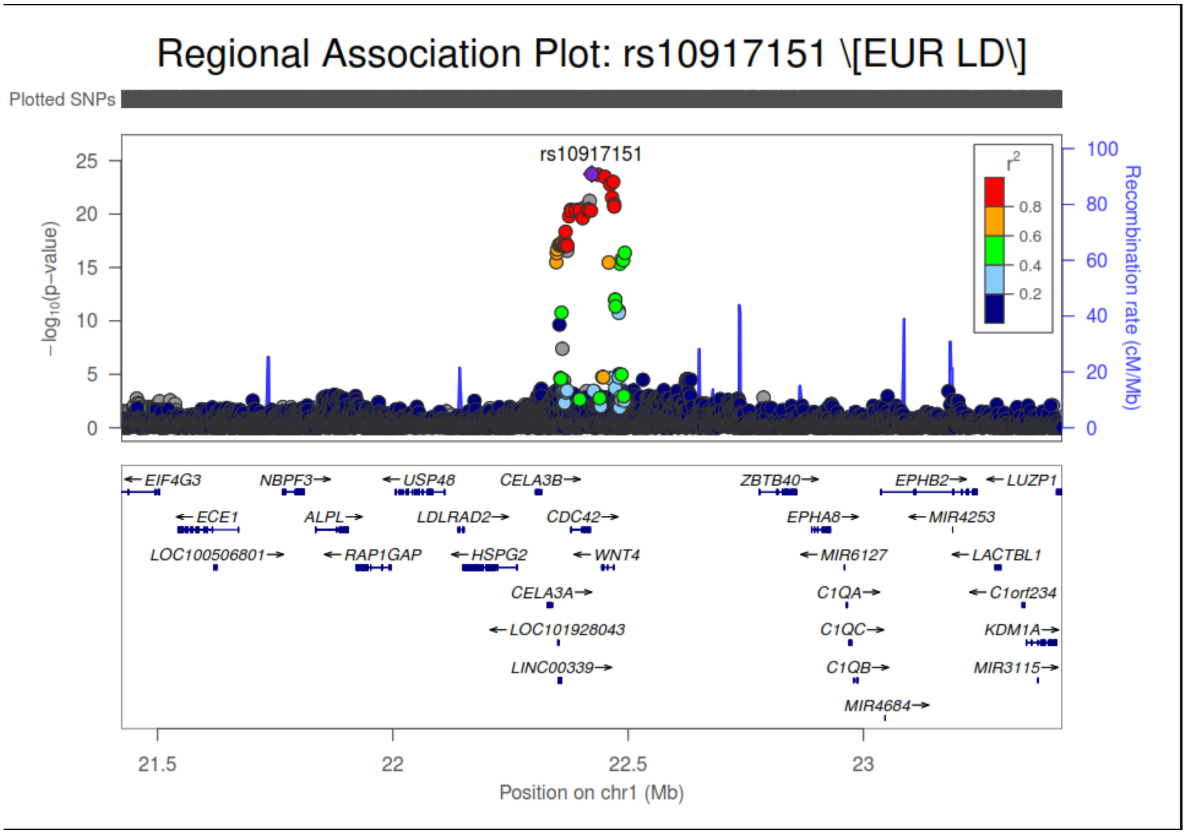

Supplementary Figures 7a-b: Regional association plots for rs58415480 from trans-ethnic final meta-analysis using the 1000 Genomes LD for African-ancestry (a) and European ancestry (b)

a

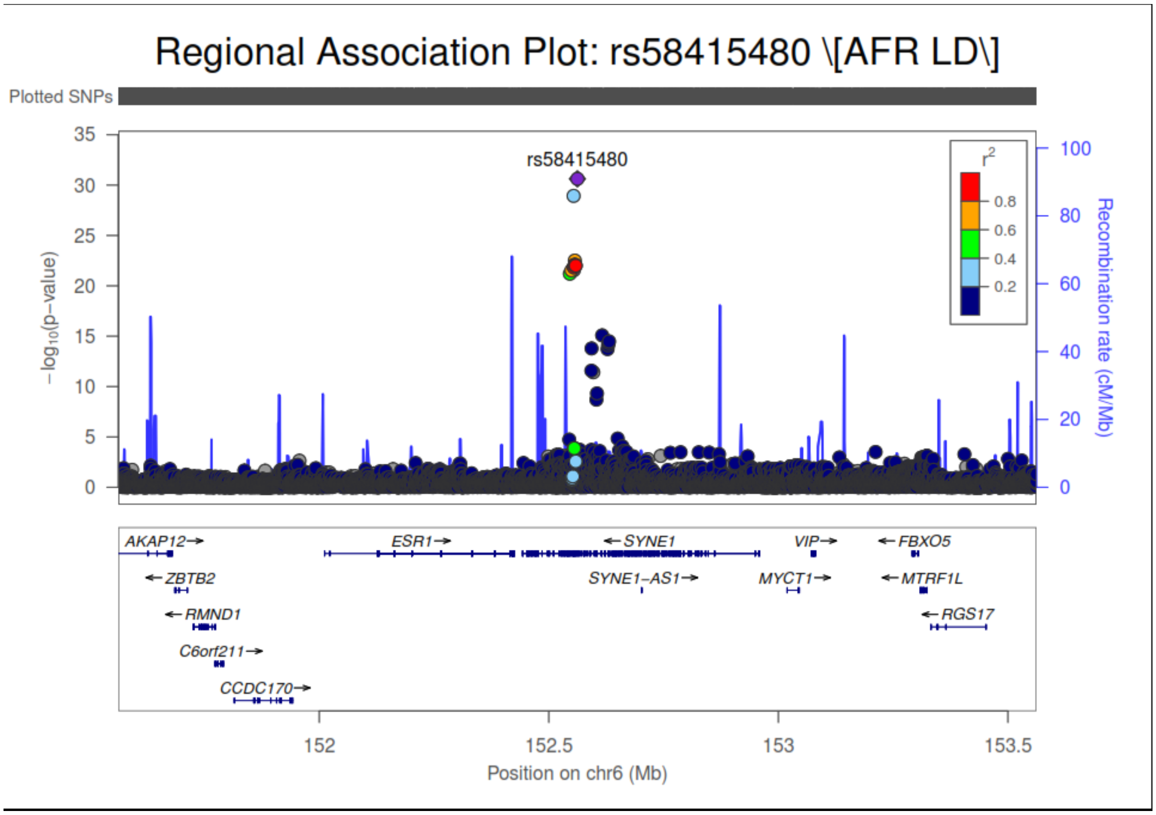

b

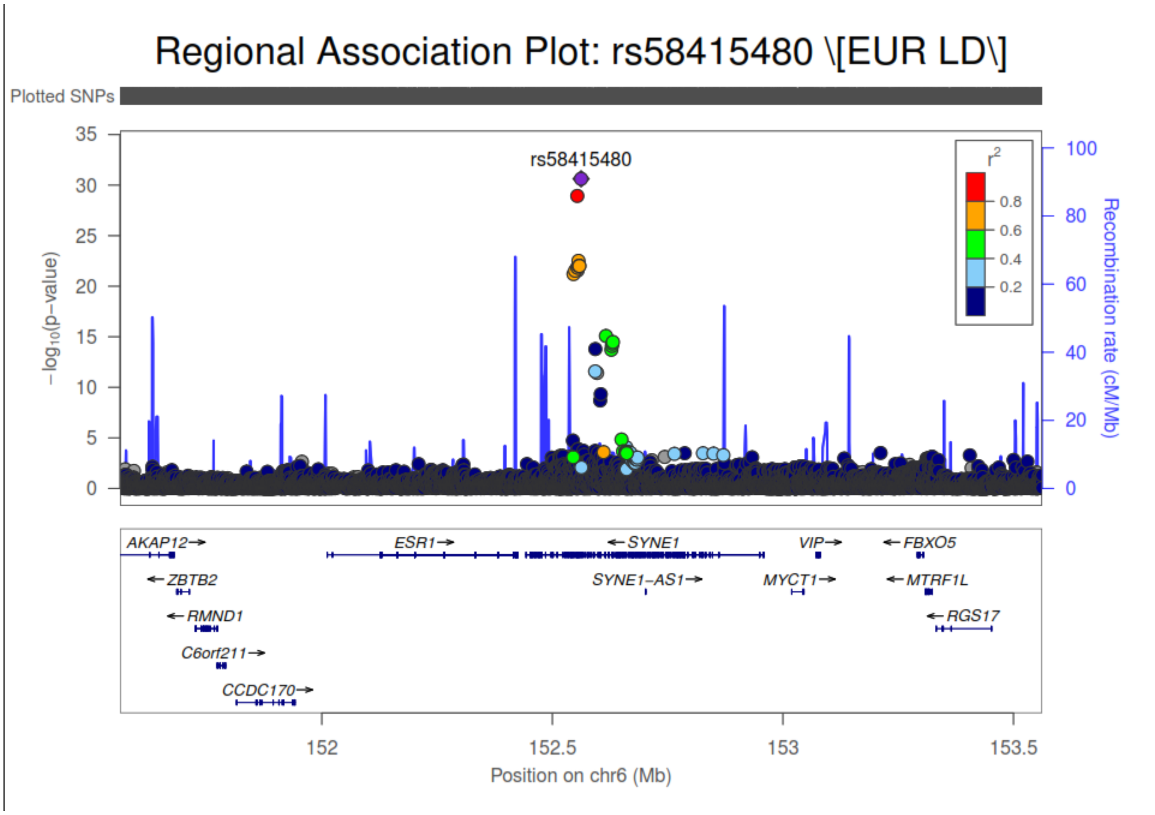

Supplementary Figures 8a-b: Regional association plots for rs1812264 from trans-ethnic final meta-analysis using the 1000 Genomes LD for African-ancestry (a) and European ancestry (b)

a

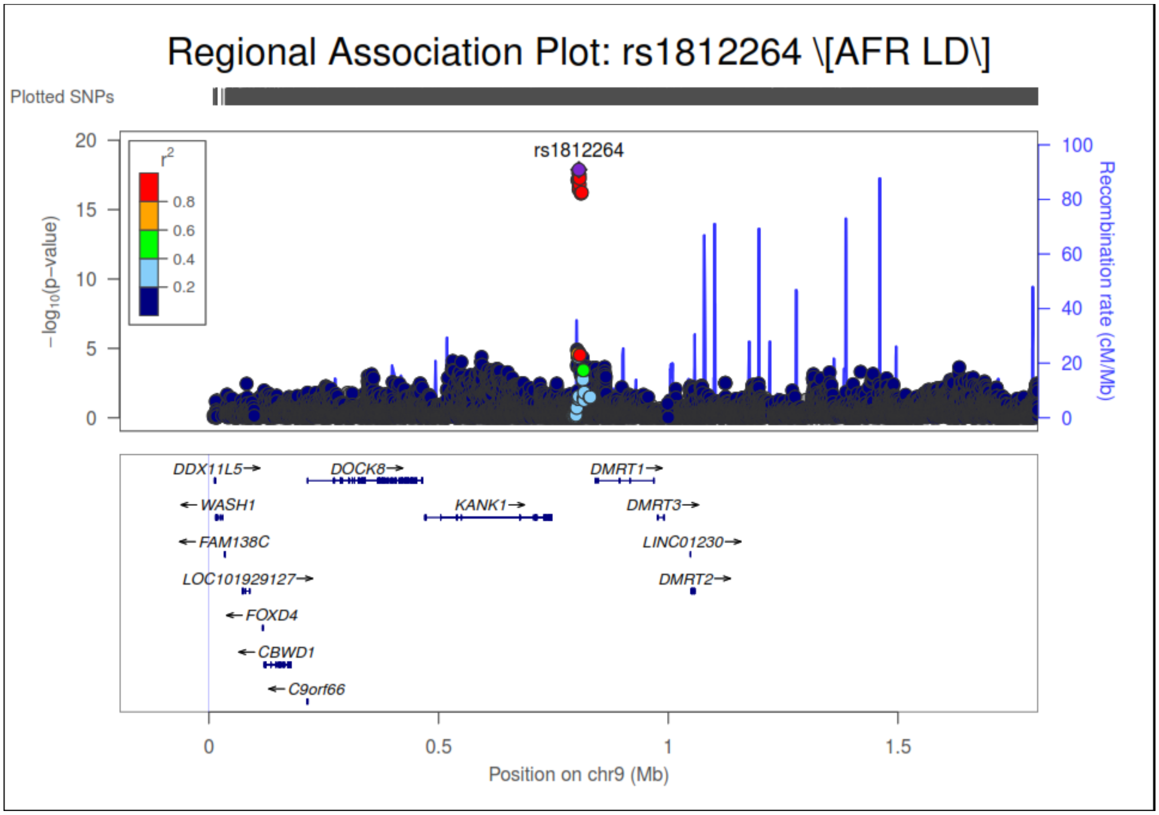

b

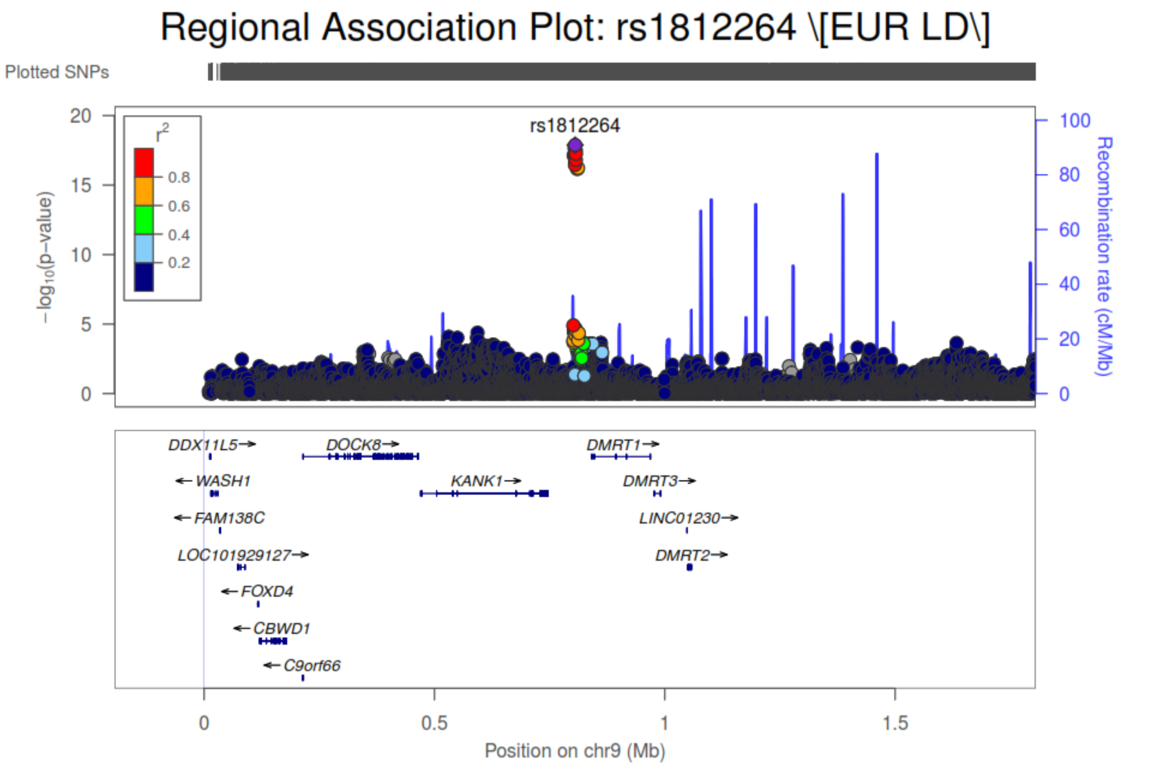

Supplementary Figures 9a-b: Regional association plots for rs7907606 from trans-ethnic final meta-analysis using the 1000 Genomes LD for African-ancestry (a) and European ancestry (b)

a

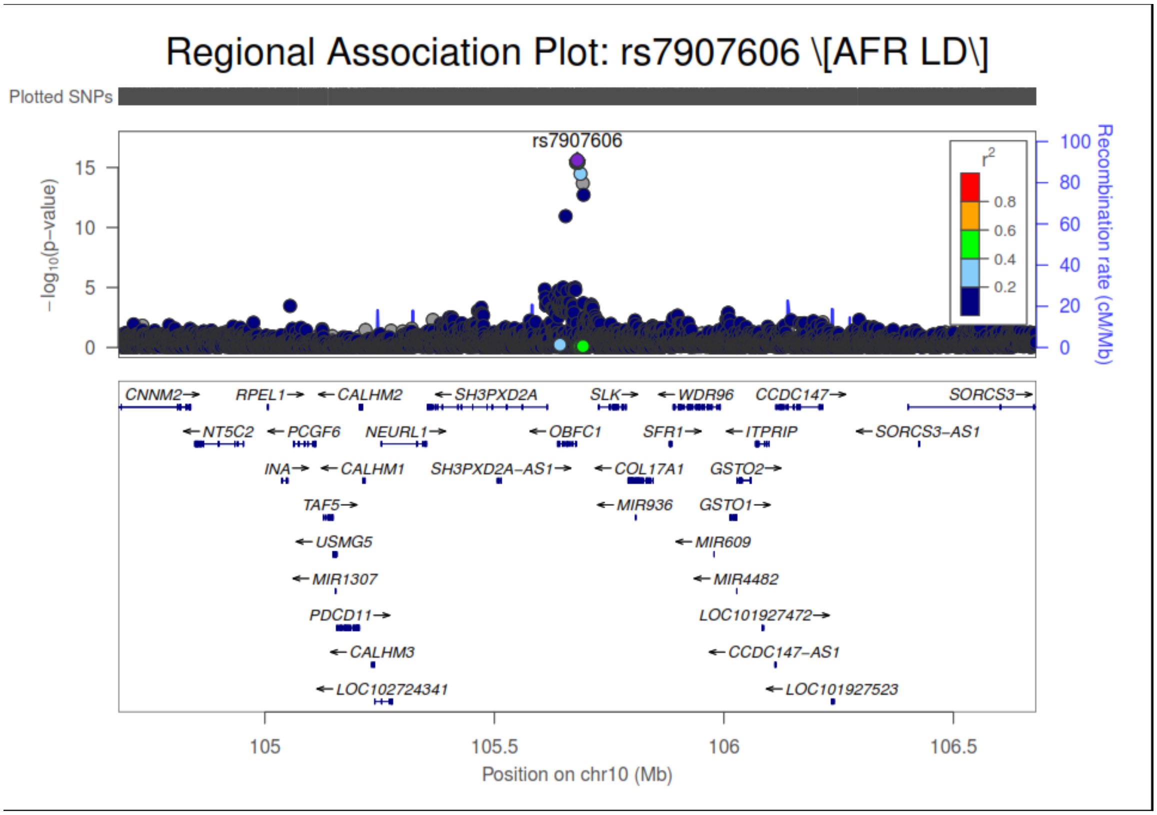

b

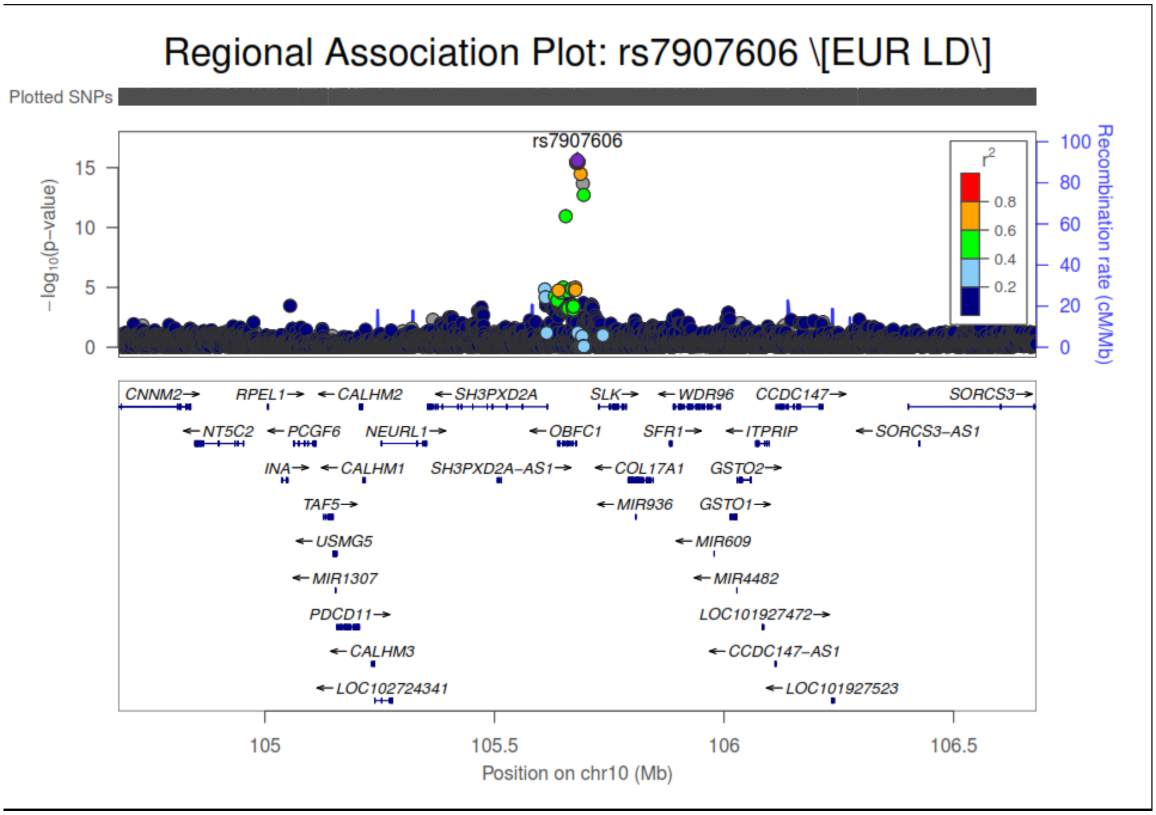

Supplementary Figures 10a-b: Regional association plots for rs7124615 from trans-ethnic final meta-analysis using the 1000 Genomes LD for African-ancestry (a) and European ancestry (b)

a

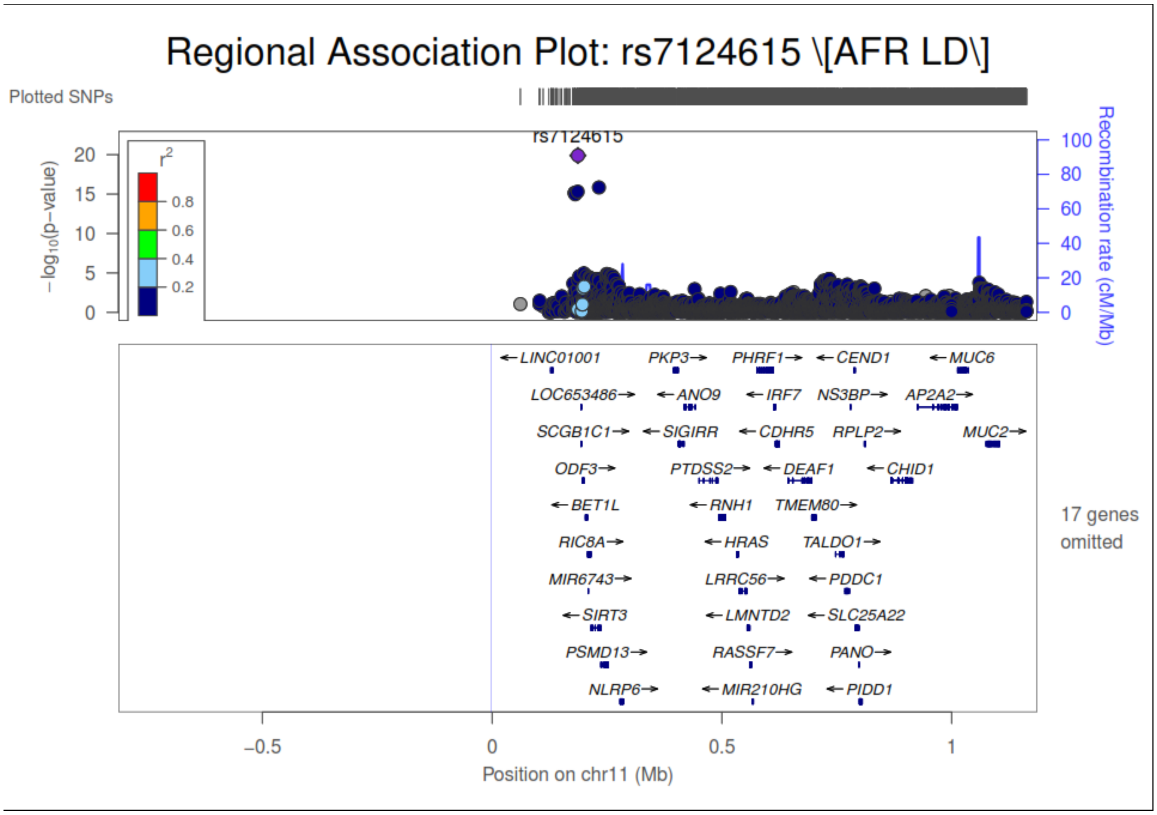

b

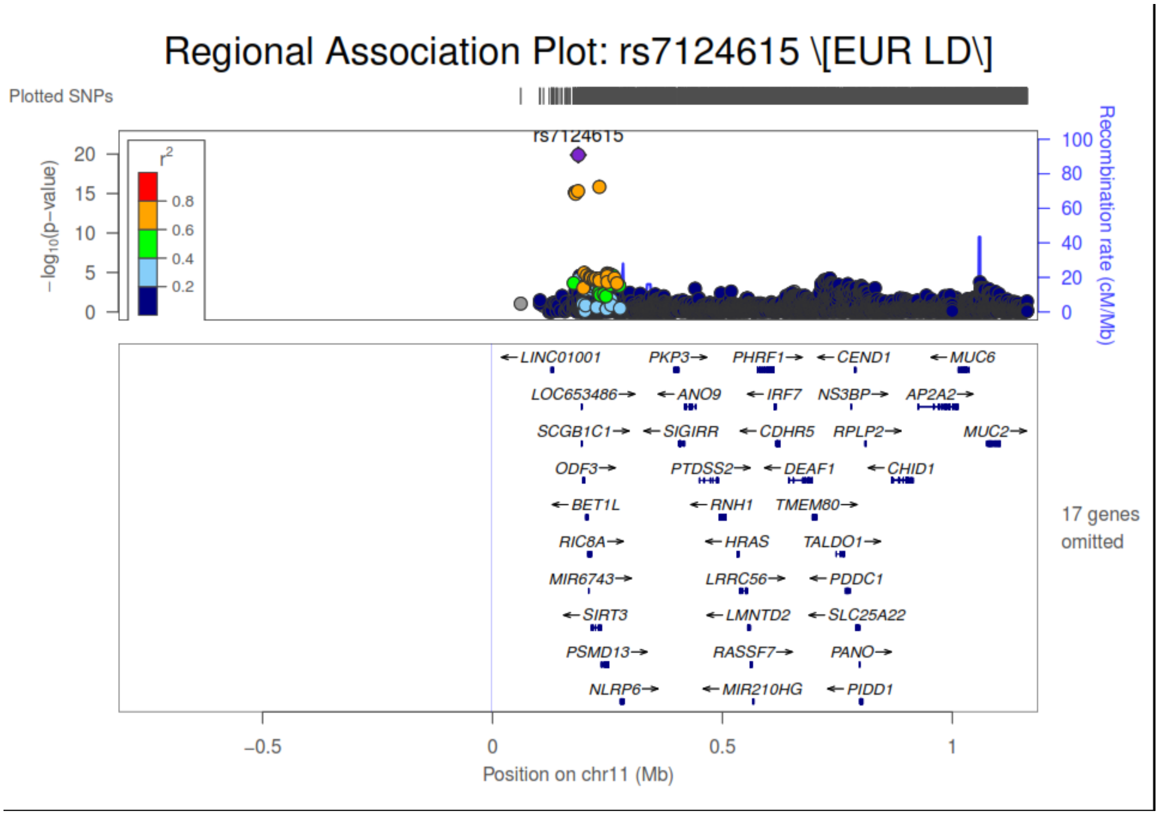

Supplementary Figures 11a-b: Regional association plots for rs10835889 from trans-ethnic final meta-analysis using the 1000 Genomes LD for African-ancestry (a) and European ancestry (b)

a

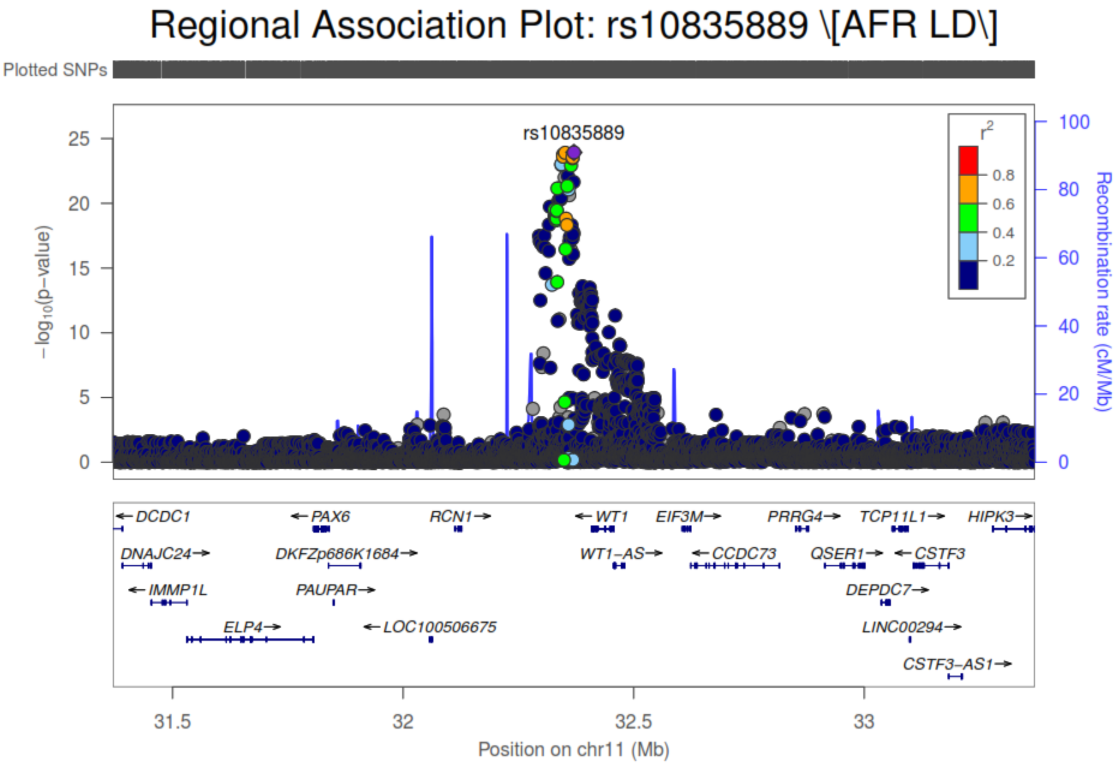

b

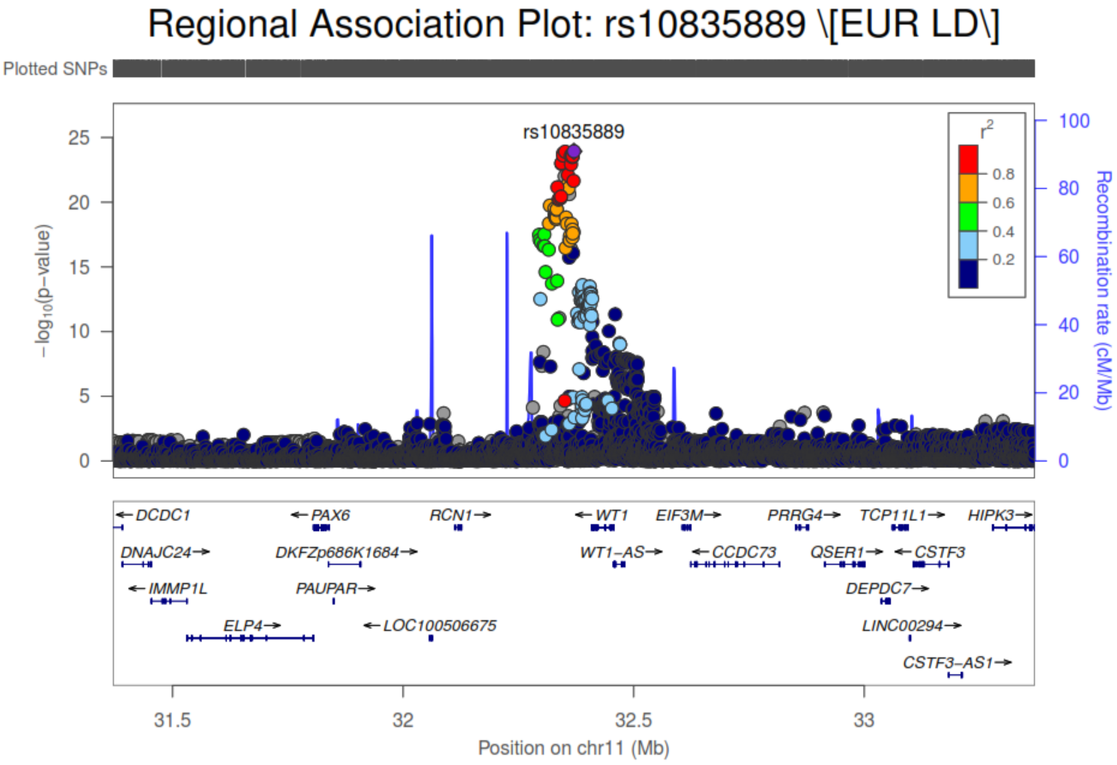

Supplementary Figures 12a-b: Regional association plots for rs78378222 from trans-ethnic final meta-analysis using the 1000 Genomes LD for African-ancestry (a) and European ancestry (b)

a

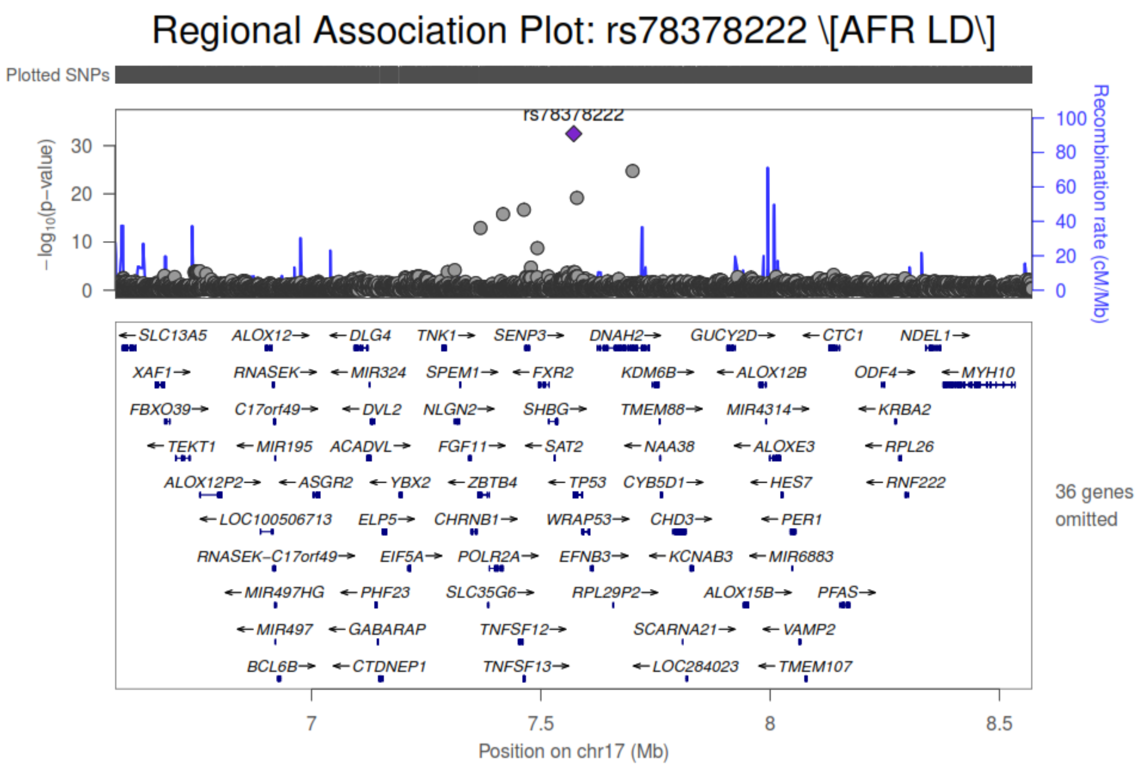

b

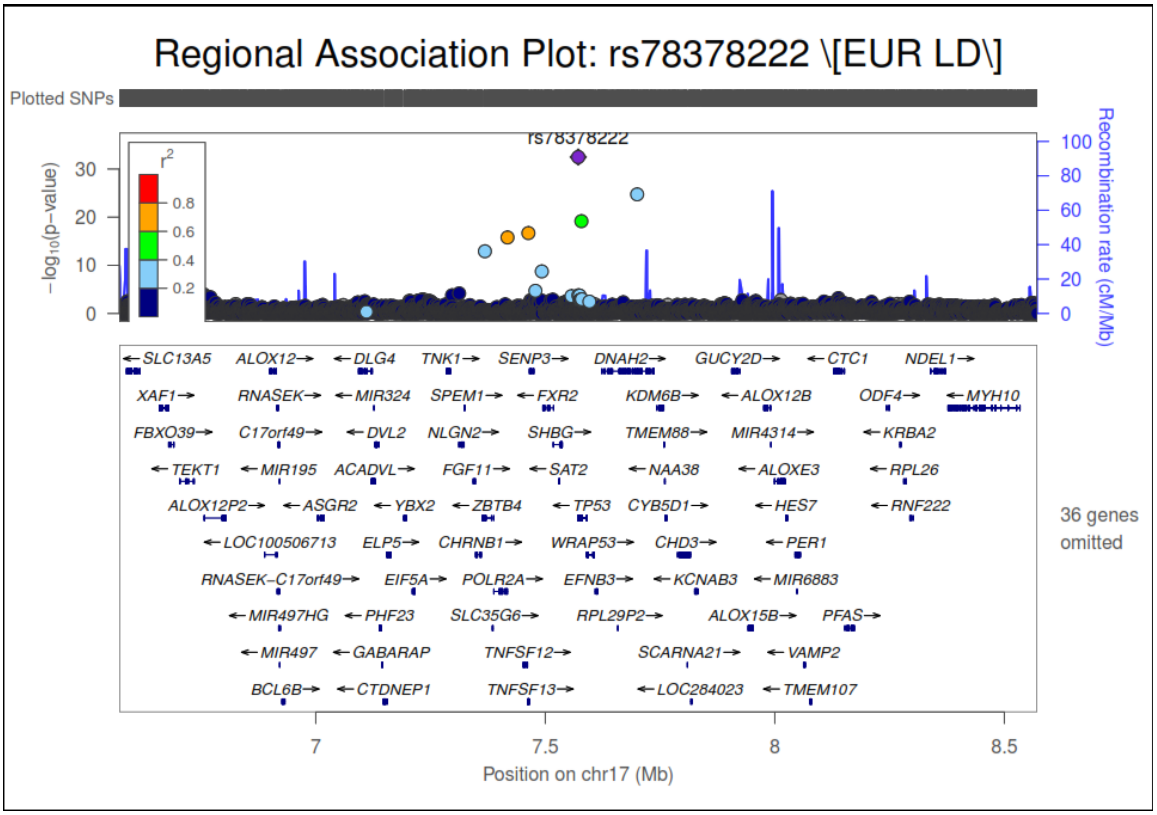

Supplementary Figures 13a-b: Regional association plots for rs3830738 from trans-ethnic final meta-analysis using the 1000 Genomes LD for African-ancestry (a) and European ancestry (b)

a

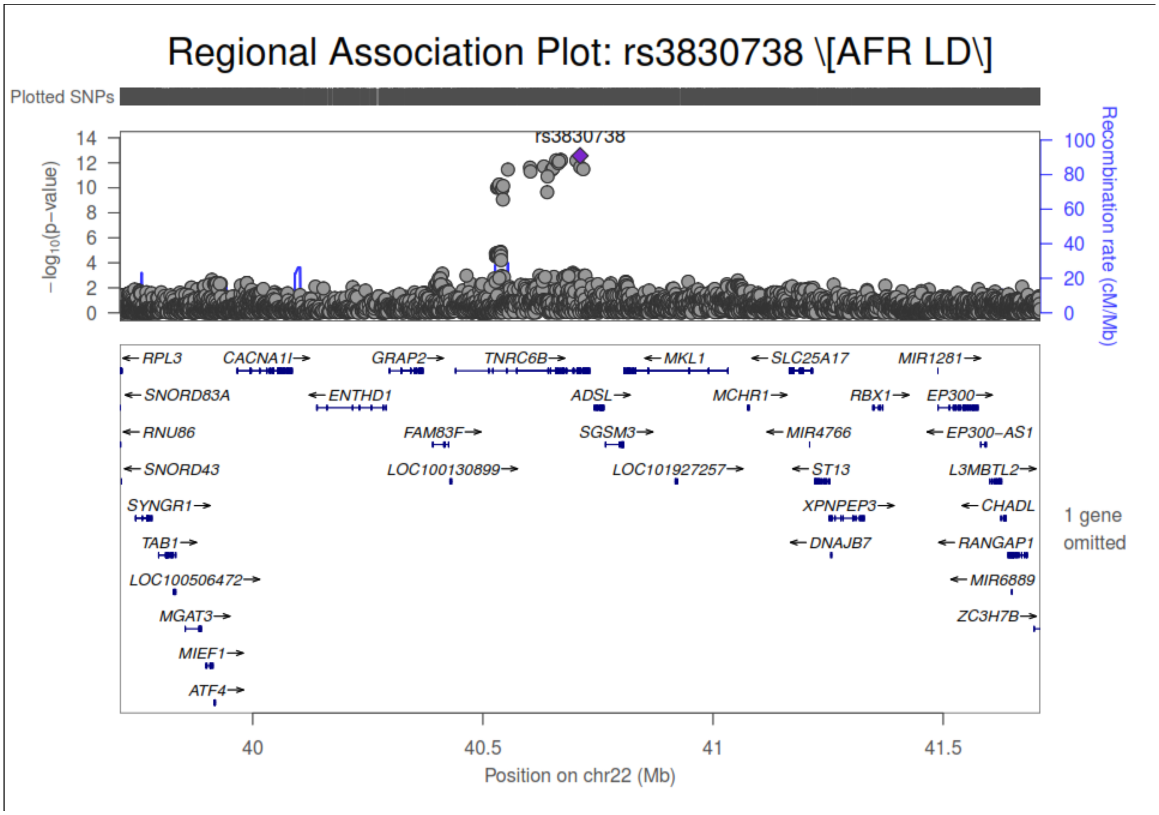

b

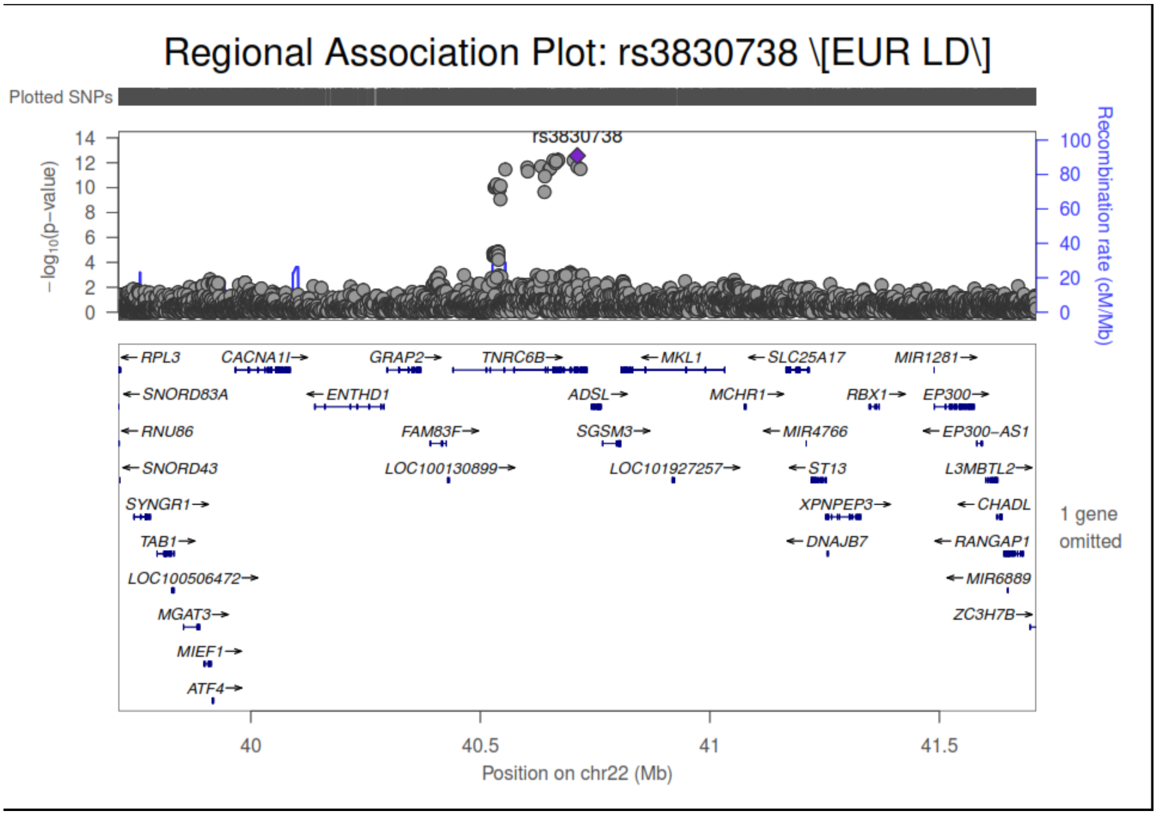

Supplementary Figure 14: Multi-tissue eQTL comparison for rs1091751 and *WNT4* gene expression in GTEx

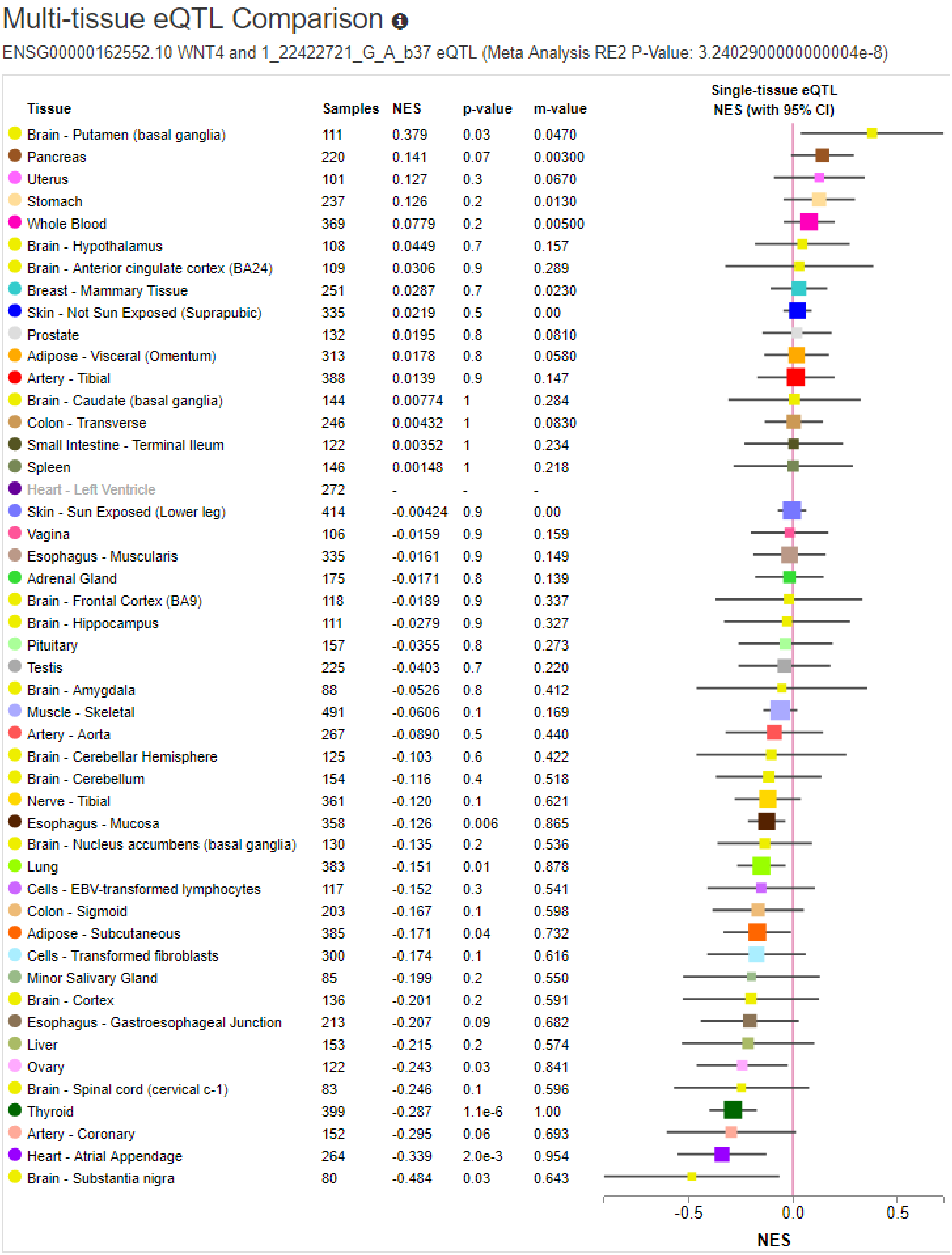
Multi-tissue eQTL Comparison

ENSG00000162552.10 WNT4 and 1_22422721_G_A_b37 eQTL (Meta Analysis RE2 P-Value: 3.24029000000000004e-8)

Tissue	Samples	NES	p-value	m-value
Brain - Putamen (basal ganglia)	111	0.379	0.03	0.0470
Pancreas	220	0.141	0.07	0.00300
Uterus	101	0.127	0.3	0.0670
Stomach	237	0.126	0.2	0.0130
Whole Blood	369	0.0779	0.2	0.00500
Brain - Hypothalamus	108	0.0449	0.7	0.157
Brain - Anterior cingulate cortex (BA24)	109	0.0306	0.9	0.289
Breast - Mammary Tissue	251	0.0287	0.7	0.0230
Skin - Not Sun Exposed (Suprapubic)	335	0.0219	0.5	0.00
Prostate	132	0.0195	0.8	0.0810
Adipose - Visceral (Omentum)	313	0.0178	0.8	0.0580
Artery - Tibial	388	0.0139	0.9	0.147
Brain - Caudate (basal ganglia)	144	0.00774	1	0.284
Colon - Transverse	246	0.00432	1	0.0830
Small Intestine - Terminal Ileum	122	0.00352	1	0.234
Spleen	146	0.00148	1	0.218
Heart - Left Ventricle	272	-	-	-
Skin - Sun Exposed (Lower leg)	414	-0.00424	0.9	0.00
Vagina	106	-0.0159	0.9	0.159
Esophagus - Muscularis	335	-0.0161	0.9	0.149
Adrenal Gland	175	-0.0171	0.8	0.139
Brain - Frontal Cortex (BA9)	118	-0.0189	0.9	0.337
Brain - Hippocampus	111	-0.0279	0.9	0.327
Pituitary	157	-0.0355	0.8	0.273
Testis	225	-0.0403	0.7	0.220
Brain - Amygdala	88	-0.0526	0.8	0.412
Muscle - Skeletal	491	-0.0606	0.1	0.169
Artery - Aorta	267	-0.0890	0.5	0.440
Brain - Cerebellar Hemisphere	125	-0.103	0.6	0.422
Brain - Cerebellum	154	-0.116	0.4	0.518
Nerve - Tibial	361	-0.120	0.1	0.621
Esophagus - Mucosa	358	-0.126	0.006	0.865
Brain - Nucleus accumbens (basal ganglia)	130	-0.135	0.2	0.536
Lung	383	-0.151	0.01	0.878
Cells - EBV-transformed lymphocytes	117	-0.152	0.3	0.541
Colon - Sigmoid	203	-0.167	0.1	0.598
Adipose - Subcutaneous	385	-0.171	0.04	0.732
Cells - Transformed fibroblasts	300	-0.174	0.1	0.616
Minor Salivary Gland	85	-0.199	0.2	0.550
Brain - Cortex	136	-0.201	0.2	0.591
Esophagus - Gastroesophageal Junction	213	-0.207	0.09	0.682
Liver	153	-0.215	0.2	0.574
Ovary	122	-0.243	0.03	0.841
Brain - Spinal cord (cervical c-1)	83	-0.246	0.1	0.596
Thyroid	399	-0.287	1.1e-6	1.00
Artery - Coronary	152	-0.295	0.06	0.693
Heart - Atrial Appendage	264	-0.339	2.0e-3	0.954
Brain - Substantia nigra	80	-0.484	0.03	0.643
